# Core outcome set for surgical trials in gastric cancer (GASTROS study): international patient and healthcare professional consensus

**DOI:** 10.1093/bjs/znab192

**Published:** 2021-06-24

**Authors:** B Alkhaffaf, A Metryka, J M Blazeby, A -M Glenny, A Adeyeye, P M Costa, I Diez del Val, S S Gisbertz, A Guner, S Law, H -J Lee, Z Li, K Nakada, D Reim, P Vorwald, G L Baiocchi, W Allum, M A Chaudry, E A Griffiths, P R Williamson, I A Bruce, the GASTROS International Working Group, S Li, Y L He, Z Xu, Y Xue, H Liang, G Li, E Zhao, P Neumann, L O'Neill, E Guinan, D Zanotti, G de Manzoni, E R C Hagens, M I van Berge Henegouwen, P Lages, S Onofre, R M Restrepo Nuñez, G Salcedo Cabañas, M Posada Gonzalez, C Marin Campos, B Candas, B Emre Baki, M Selim Bodur, R Yildirim, A Burak Cekic, J Brown, K Hayes, I Daher, R H Gianchandani Moorjani, A Adetoyese Adeyeye, A Sulaiman Olayide, A Mitsuo Leon-Takahashi, A Pueyo Rabanal, A Peri, A Boddy, A Novotny, A Charalabopoulos, A Alemdar, A Souadka, A M Rodrigues Gomes, A Lázaro, A Maciel Da Silva, A do Rosário da Conceição Silva e Santos, A Guidi, A J Silva Bernardes, A Quinn, A Isik, A A Slipek, B Candaş, Altinbaş, B Johnson Alegbeleye, B Wool Eom, B Frittoli, B Lonsdale, B Rogers, B J Ammori, B Rau, B Molteni, B E Byrne, B A Villacís-Bermeo, B E Villacís Gallardo, B Köse, C J Sampedro Nogueira, C Loureiro, C M Oliveira de Sousa, C G Collins, C Nonso Ekwunife, C Chukwunwendu Osuagwu, C L.-Y Wong, C Winkler, D Reim, D W Kjær, D Cooper, D Horner, D Irvine, D J Bowrey, D J Chuter, D Elliot, D McGhee, D Toth, D Öfner, D K Manatakis, D R Silveira Martins, E J T Belt, E Cattaneo, E Samadov, E Colak, E Treppiedi, E Guglielmi, E Redondo-Villahoz, E Ciferri, E Tiemens-de Graaf, E Cocozza, E Pape, E S Drozdov, F Enrico, F Rashid, F Marco, F Rosa, F Mingol Navarro, F Simionato Perrotta, F S.-Y Chan, F D Saavedra Tomasich, F R Takeda, F Farrell, F Olanike Wuraola, G Rosero, G Bevilacqua, G Baronio, G Mura, G de Manzoni, G D'Eugenio, G Ortega-Perez, G Tilt, G Sutcliffe, G Mureddu, G Guerra Jacob, G H Daneri, H Olufemi Gbenga, H Okabe, I Kingsford Smith, I Olawale Lateef, I Garosio, İ Hatipoğlu, I Gockel, I Negoi, I S.-H Min, I M M Mesquita, I Diez del Val, J H F Leemhuis, J A Gossage, J Weindelmayer, J R Izbicki, J McKenzie Manson, J Kelly, J H M B Stoot, J W Haveman, J D Brown, J Sultan, J Hassall, J van Sandick, J H Saunders, J K Clarke, J Heisterkamp, J I Vargas R, J M Couselo Villanueva, J Ingmire, J McEwen, J Galindo Álvarez, J Turner, J Peng, K Roberts, K G Brandon, K Mitchell, K McCarthy, K Akhtar, K N Mikhailovich, L Corbelli, L Medeiros Milhomem, L Solaini, L Fengyuan, L Xinchun, L Timmermans, L Porritt, L Taglietti, L Bonavina, L F Pinheiro, M de los Angeles Mayo Ossorio, M Schiavo, M Marchesiello, M das Dores Vieira Leite, M DeMois, M Posada Gonzalez, M T Di Felice, M I van Berge Henegouwen, M D de Sousa, M Takahashi, M Forshaw, M Berselli, M Paro, M A Usta, M.-H Yan, M Pinchin, M CapriolI, M Rubbini, M Cowen, M A Herrera Servin, M.-Z Li, M Sasako, M Shukri Jahit, M Ngonyoku Muhinga, M A Tareen, M F Ahmad, M S Bodur, M Kaban, N Farooq, N Coburn, N Cooper, N S Blencowe, N Loria, N de Vries, N Adami Andreollo, N Köksal, N Zanini, N Kreuser, N Okkabaz, O Damiana, O Afuwape, O Kayode Fasiku, O Comensoli, O F Koroye, P Capener, P Morgagni, P M Pernadas Lages, P M Wilkerson, P Turner, P Dutton, P Hayes, P Vorwald, P Singh, Q Gan, R Kottayasamy Seenivasagam, R Ayloor Seshadri, R Guevara Castro, R Douglas, R M Koshy, R Yıldırım, R J E Skipworth, R A Gould, R C Wetherill, R Shaw, R A Burley, R Palatucci, R Racalbuto, R M Correia Casaca, S M Lagarde, S Gana, S Marietti, S Qureshi, S Morales-Conde, S Molfino, S G Barreto, S Turkyilmaz, S Turan-Trabzon, S Frisch, S Castoldi, S Belloni, S Flisi, S Galloway, S R Maria, S Royston, T Boyle, T Ö Sezer, V Mengardo, V Concepción Martín, V Lee Wills, V Owen-Holt, V Casagrande, W Al-Khyatt, W Jansen, W Wang, W Eshuis, W P Polkowski, X Huang, X Wang, X.-Z Chen, Y Gonzalez Dominguez, Y Wang, Y K S Viswanath, Y.-L He, Z Demir, Z Na

**Affiliations:** Department of Oesophago-Gastric Surgery, Salford Royal Hospital, Salford Royal NHS Foundation Trust, Salford, UK; Division of Cancer Sciences, School of Medical Sciences, Faculty of Biology, Medicine and Health, University of Manchester, Manchester, UK; Paediatric Ear, Nose and Throat Department, Royal Manchester Children’s Hospital, Manchester University NHS Foundation Trust, Manchester, UK; Centre for Surgical Research and Bristol and Weston National Institute for Health Research Biomedical Research Centre, University of Bristol, Bristol, UK; Division of Dentistry, School of Medical Sciences, Faculty of Biology, Medicine and Health, University of Manchester, Manchester, UK; University of Ilorin Teaching Hospital, Ilorin, Nigeria; Cirurgia Geral, Hospital Garcia de Orta, Faculdade de Medicina da Universidade de Lisboa, Lisbon, Portugal; Basurto University Hospital, Bilbao, Spain; Department of Surgery, Cancer Centre, Amsterdam UMC, University of Amsterdam, Amsterdam, the Netherlands; Department of General Surgery, Faculty of Medicine, Karadeniz Technical University, Trabzon, Turkey; Department of Surgery, University of Hong Kong, Hong Kong, China; Department of Surgery and Cancer Research Institute, Seoul National University College of Medicine, South Korea; Peking University Cancer Hospital and Institute, Beijing, China; Department of Laboratory Medicine, Jikei University School of Medicine, Tokyo, Japan; Department of Surgery, TUM School of Medicine, Munich, Germany; Hospital Universitario Fundación Jiménez Diaz, Madrid, Spain; Department of Clinical and Experimental Sciences, University of Brescia, Brescia, Italy; Department of Academic Surgery, Royal Marsden NHS Foundation Trust, London, UK; Department of Academic Surgery, Royal Marsden NHS Foundation Trust, London, UK; Department of Upper Gastrointestinal Surgery, University Hospitals Birmingham NHS Foundation Trust, Queen Elizabeth Hospital Birmingham, Birmingham, UK; Medical Research Council North West Hub for Trials Methodology Research, University of Liverpool, Liverpool, UK; Division of Cancer Sciences, School of Medical Sciences, Faculty of Biology, Medicine and Health, University of Manchester, Manchester, UK; Division of Infection, Immunity and Respiratory Medicine, Faculty of Biology, Medicine and Health, University of Manchester, Manchester, UK

## Abstract

**Background:**

Surgery is the primary treatment that can offer potential cure for gastric cancer, but is associated with significant risks. Identifying optimal surgical approaches should be based on comparing outcomes from well designed trials. Currently, trials report different outcomes, making synthesis of evidence difficult. To address this, the aim of this study was to develop a core outcome set (COS)—a standardized group of outcomes important to key international stakeholders—that should be reported by future trials in this field.

**Methods:**

Stage 1 of the study involved identifying potentially important outcomes from previous trials and a series of patient interviews. Stage 2 involved patients and healthcare professionals prioritizing outcomes using a multilanguage international Delphi survey that informed an international consensus meeting at which the COS was finalized.

**Results:**

Some 498 outcomes were identified from previously reported trials and patient interviews, and rationalized into 56 items presented in the Delphi survey. A total of 952 patients, surgeons, and nurses enrolled in round 1 of the survey, and 662 (70 per cent) completed round 2. Following the consensus meeting, eight outcomes were included in the COS: disease-free survival, disease-specific survival, surgery-related death, recurrence, completeness of tumour removal, overall quality of life, nutritional effects, and ‘serious’ adverse events.

**Conclusion:**

A COS for surgical trials in gastric cancer has been developed with international patients and healthcare professionals. This is a minimum set of outcomes that is recommended to be used in all future trials in this field to improve trial design and synthesis of evidence.

## Teaser

Outcomes in surgical trials for gastric cancer are reported heterogeneously and do not sufficiently reflect the priorities of key stakeholders, including patients. This contributes to outcome reporting bias and research waste. To address these challenges, a core outcome set—a minimum group of critically important outcomes to be reported in future trials— has been developed through an international consensus process involving patients and healthcare professionals.

## Introduction

Gastric cancer is a significant global health burden which is associated with poor survival^1^. Although the adoption of multimodal therapy for the minority of patients who present with early-stage disease has improved prognosis, surgery remains the only treatment offering a potential cure^2^. Identifying the optimum surgical approach involves balancing the benefits of a radical oncological resection against the risk and impact of associated complications and physiological consequences. The ability to compare outcomes from surgical trials in a clinically meaningful manner is crucial to this process.

Homogeneity in the selection and reporting of key outcomes between studies is necessary if useful synthesis of evidence is to be achieved. However, outcome reporting in surgical trials for gastric cancer is heterogeneous and not based on methodologically robust standards^3^. Even when similar outcomes are reported, different definitions, measurement instruments, and time points are used. Likewise, patient priorities and perspectives tend to be overlooked when outcomes are selected by researchers. This potentially limits the subsequent relevance of aspects of the research effort to the most important stakeholder group^4^. For example, quality of life, an area identified as vitally important to patients, is reported in less than 10 per cent of trials^3^.

To address this challenge, the GASTROS study (GAstric cancer Surgery Trials Reported Outcomes Standardization) was undertaken to develop a core outcome set (COS) for surgical trials in gastric cancer^5^. A COS is ‘an agreed, standardized collection of outcomes which should be measured and reported, as a minimum, in all trials for a specific clinical area’^6^. Outcomes should be relevant to key stakeholders, who should contribute to the stages of COS development.

These challenges in outcome reporting are not limited to the field of gastric cancer and affect virtually all clinical areas. COMET (Core Outcome Measures in Effectiveness Trials; https://www.comet-initiative.org) is an initiative that aims to promote COS development^7^. Its registry database and up-to-date systematic reviews have comprehensively mapped COS projects across all disciplines^8–12^. Although groups have developed COSs for different gastrointestinal cancers^13–15^, there has yet to be one developed for gastric cancer. The global incidence of gastric cancer and differences in patient characteristics, management, and outcomes, necessitated an international approach to this COS^16^^,^^17^. An International Working Group (IWG) of collaborators was set up to support this project, aided by a comprehensive network of patient organizations, charities, and professional bodies across six continents.

## Methods

The GASTROS study conforms to standards established for the development of COS as outlined by COS-STAD (Core Outcome Set—STAndards for Development)^18^. This report uses the COS-STAR (Core Outcome Set—STAndards for Reporting) standards to describe the development of a COS for surgical trials in gastric cancer^19^. The checklist is provided in *Table S1*.

### Scope

**Fig. 1:**
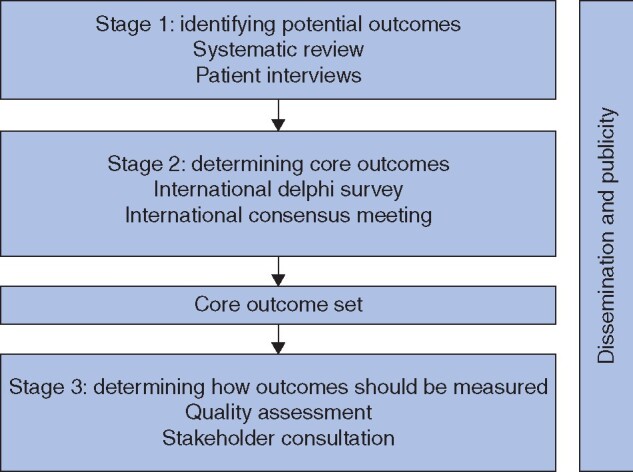
Stages of the GASTROS study

The scope, objectives, and methodological approaches of this study have been described previously in detail^5^. In summary, the COS developed in this study was aimed at all clinical effectiveness trials examining therapeutic surgical trials for patients with early-stage (potentially curable) gastric cancer. The GASTROS study used existing best-practice approaches as developed by the COMET Initiative, while adapting the methodological principles to the challenges of an international consensus exercise. An overview of the study stages is shown in *Fig. 1*. This publication describes stages 1 and 2.

### Stakeholder participants and eligibility

The GASTROS study aimed to consider the views of key stakeholders during the process, namely patients, surgeons, and oncology nurses. The guiding principle was to promote the patient voice as they are the beneficiaries of trials in this field and have all-important lived experience. Surgeons provide a clinical perspective and the experience of treating large volumes of patients. Oncology nurses were invited to participate given their central roles as caregivers, patient advocates, and core members of the clinical team. Participation in the study was open to all interested stakeholders who fulfilled the following criteria: surgeons who had completed their training and routinely treat gastric cancer, oncology nurses with a recognized proportion of their role involved in the care and follow-up of patients with gastric cancer, and patients who have undergone surgical resection for gastric cancer with the intention of cure. Patients and healthcare professionals were identified through local, regional, and national clinical and research networks. Support from patient groups, charities, and professional societies was also key.

### Stages of GASTROS study

Stage 1 (*Fig. 1*) identified outcomes that may be important to stakeholders and stage 2 subsequently prioritized outcomes for inclusion in the final COS (what to measure). In addition, the GASTROS study aimed to collate the corresponding outcome measurement instruments used in surgical trials and to determine the variability in measurement time points, for use in future outcomes research (to determine how and when to measure) in gastric cancer.

#### Stage 1: identifying outcomes

A systematic review of surgical trials for gastric cancer over two decades was undertaken from which all reported outcomes were extracted verbatim^3^. Patient-reported outcome measurement instruments used in these trials were broken down into their component parts to identify additional outcomes. To ensure that patients’ perspectives were captured, a series of qualitative interviews exploring outcome prioritization was undertaken to identify potentially important outcomes not identified in the systematic review^4^. A subsequent long list of potentially important outcomes was compiled which underwent a process of rationalization, before being presented to the stakeholder groups for prioritization in stage 2.

The rationalization process (*Table S2*) was initiated through discussion within the Study Management Group (SMG) to merge closely related items and map them against a taxonomy developed for COSs^20^. This process was assessed independently by an external methodologist with extensive experience in COS development. The resulting short list was presented to stakeholder representatives (patients, surgeons, and oncology nurses) comprising the Study Advisory Group (SAG). The SAG was tasked with ratifying the process so far, further merging of outcomes if required, developing plain-language descriptions of the outcomes, and identifying additional outcomes that they believed had not yet been identified. Following this, the short list of outcomes and corresponding plain-language descriptions were presented to a patient group as part of a cognitive debriefing exercise to ensure understanding and comprehensibility.

#### Stage 2: prioritizing outcomes

To prioritize which items to include in the COS, patients, surgeons, and oncology nurses were invited to participate in an international, multilanguage Delphi survey. The methodological approach used to translate the surveys and recruit participants has been described separately^21^. Although there is no formal sample size requirement for Delphi surveys, recruitment was facilitated with the support of a large network of professional bodies, patient groups, and charities, to help ensure that a representative spectrum of opinion was captured for each stakeholder group.

Participants were invited to score outcomes in terms of importance on a Likert-type scale ranging from 1 to 9 (1–3, not important; 4–6, important; 7–9, critically important) in a two-round online Delphi survey. Participants were given the opportunity to add outcomes they considered were missing, for consideration by participants in round 2. Suggested additional outcomes were considered by the SMG and reviewed independently by an external methodologist with experience of cancer-related surgical COSs. The scores of each stakeholder group were collated and summarized separately to ensure an equal voice among stakeholder groups. Participants who had completed 50 per cent of the first survey were included in the round 1 analysis and invited to participate in round 2. They were then presented with group scores (presented as score distribution charts) for each stakeholder group from round 1, and given the opportunity to reflect on the opinions of others before deciding whether to change their scores for each outcome in round 2. Those who changed scores between rounds were able to provide a reason for this^18^. After two rounds of voting, outcomes were categorized according to predetermined criteria for inclusion in, or exclusion from, the COS. Participants who had completed at least 50 per cent of the survey in round 2 were included in the final analysis.

Any outcome scored as critically important (7–9) by more than 70 per cent and not important (1–3) by less than 15 per cent in all three stakeholder groups was categorized for inclusion. Any outcome scored as critically important (7–9) by less than 50 per cent in all three stakeholder groups was categorized for exclusion. These criteria were adapted from established COS methodology^6^. Outcomes achieving any other combination of scores were categorized as not having reached consensus (no consensus) and presented for further discussion at a consensus meeting.

Survey participants were invited to attend a consensus meeting in Manchester (UK) during March 2020. The aim of the meeting was to review the results of the Delphi survey and consider the outcomes for which no consensus was reached before finalizing the COS. Participants could take part by attending the meeting venue in person, or through an online platform. The meeting was undertaken in English and chaired by a clinical academic from the SMG with experience in COS development, and with no clinical expertise in the management of gastric cancer.

Following discussion, stakeholders were asked to score outcomes using the same criteria as was set out in the Delphi survey. Similarly, scores from each stakeholder group were considered separately to mitigate for imbalance in the numbers of each participant type. Turning Point software (Turning Technologies, Youngstown, OH, USA) was used to support voting at the venue and online simultaneously. Participants were also asked to complete an online voting form to mitigate against software malfunction. Outcomes reaching the original consensus criteria for inclusion in the final COS were to be added to those included from the Delphi survey.

### Assessing bias

To assess the impact of attrition bias between survey rounds, mean scores of participants completing both rounds of the Delphi survey were compared against those of participants completing round 1 alone. Mean scores of those who took part in the Delphi survey but did not attend the consensus meeting were compared against the mean scores of those who attended to assess the degree to which consensus meeting participants were representative of those who participated in the survey. Both analyses were undertaken using a *t* test to examine for statistically significant differences at the 0.05 level. Furthermore, the characteristics of stakeholders participating in both rounds were compared with those who only completed round 1. A descriptive analysis was undertaken, and the c^2^ test applied to examine for statistically significant differences at the 0.05 level.

### Patient and public involvement

A guiding principle of the GASTROS study was that patients’ voices should be represented at each stage of the project. Patient representatives were integral with membership in the SAG and support from international charities. The dissemination of results from this study will be supported by a network of international charities and patient support groups. The patient representatives opted to participate in the patient-focused study report of results rather than the scientific dissemination.

### Study registration and protocol

The GASTROS study was registered in the COMET database (http://www.comet-initiative.org/studies/details/764?result=true) before commencement. The study protocol has been described previously^5^.

### Ethical approvals and portfolio adoption

Ethical approvals were required for the qualitative patient interviews and international Delphi surveys. The qualitative interview study was given ethical approval by the National Research Ethics Service North West—Cheshire (11/NW/0739) and governance approvals by Central Manchester University Hospital National Health Service (NHS) Foundation Trust. The Delphi survey was given ethical approval by the North West—Greater Manchester East Research Ethics Committee (18/NW/0347) and governance approvals by Manchester University Hospitals NHS Foundation Trust. Both the patient interviews and Delphi survey were adopted on to the National Institute for Health Research (NIHR) Clinical Research Network Portfolio (IDs 33312 and 38318). Ethical approval for international participants was sought and obtained locally by IWG collaborators.

## Results

**Fig. 2:**
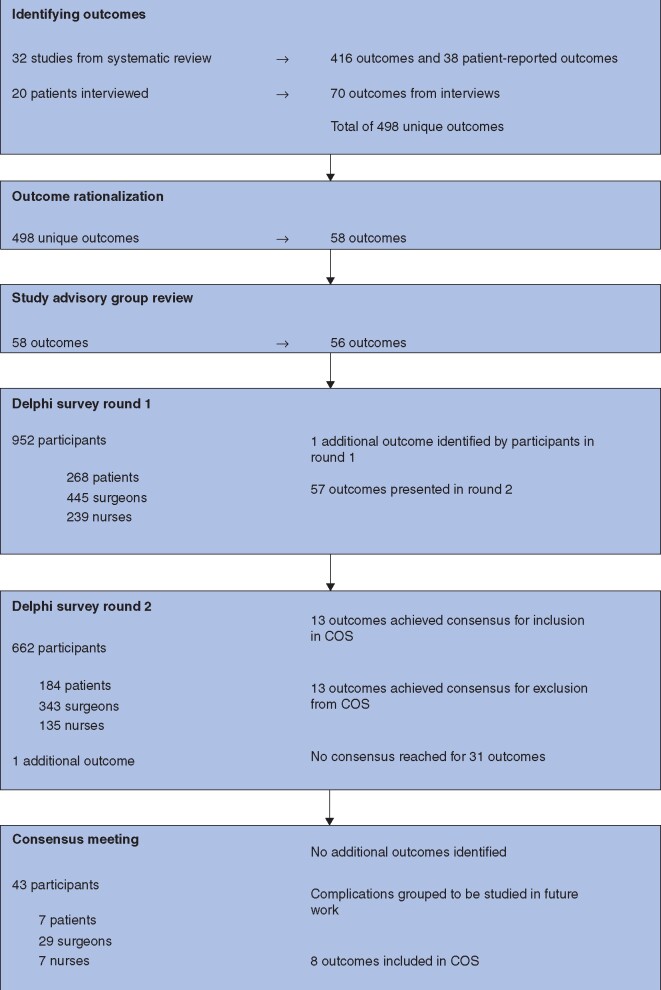
Results from different stages of the GASTROS study COS, core outcome set.

The results for each stage of the study are summarized in *Fig. 2*. The 498 outcomes identified from the systematic review, patient-reported outcome measures, and patient interviews were rationalized by the SMG into 58 items, which were presented to the SAG. The SAG merged chyle leak, nutritional complications, respiratory function, surgical-site infection, and time to ambulation into other existing outcomes. Bleeding, anaesthetic complications, and destination on discharge were expanded or added as separate outcomes, which meant that a total of 56 items were presented to participants in the Delphi survey (*Table S3*).

### Delphi survey

A total of 1021 patients, surgeons, and oncology nurses registered for the Delphi survey, of whom 952 (268 patients, 445 surgeons, 239 nurses) from 55 countries across six continents fulfilled the criteria for inclusion in the round 1 analysis. *Table 1* summarizes the characteristics of those included in the analyses. *Table S4* details the results of voting in both rounds. One additional outcome (duration of stay in an intensive care ward) suggested by participants in round 1 was presented in round 2 along with the original 56 outcomes for rescoring (a total of 57 outcomes in round 2). Although other outcomes were suggested by participants in round 1, these were deemed by the SMG and an external reviewer as either direct duplication of outcomes already included or not sufficiently unique that they warranted being presented separately (*Table S5*). Scores from 662 participants in round 2 were included in the final analysis representing an attrition rate of 30 per cent. Some 557 participants (84 per cent) changed the score of least one answer from round 1, with 191 (29 per cent) participants changing a score to cross a boundary (for example, from 1–3 to 4–6 or 7–9). A detailed analysis exploring the reasons for changing scores has been reported previously^18^.

**Table 1:** Demographic characteristics of participants included in analysis of round 1 and 2 scores

Stakeholder group	Variable	**Completed round 1 only** **(**%**)**	**Completed both rounds** **(%)**	***P*** ^*^
**Patients (*n =* 268)**	**All**	84	184	
	**Age (years)**			0.602
	<60	38 (45)	77 (42)	
	≥60	46 (55)	107 (58)	
	**Sex**			0.345
	M	52 (62)	101 (55)	
	F	32 (38)	83 (45)	
	**Region**			0.185
	West	53 (63)	113 (61)	
	East	23 (27)	39 (21)	
	Other	8 (10)	32 (17)	
	**Country income**			0.792
	HIC	53 (63)	113 (61)	
	LMIC	31 (37)	71 (39)	
	**Time since surgery (years)**			0.656
	<1	15 (19)	30 (17)	
	1–3	34 (44)	68 (39)	
	>3	29 (37)	75 (43)	
	**Surgical approach**			0.850
	Open	70 (83)	145 (78)	
	Minimally invasive	14 (17)	31 (22)	
	**Type of gastrectomy**			0.503
	Total	40 (49)	78 (44)	
	Partial	42 (51)	98 (56)	
	**Treatment modality**			0.495
	Surgery alone	28 (34)	69 (39)	
	Multimodal therapy	54 (66)	110 (61)	
**Surgeons (*n* = 445)**	**All**	102	343	
	**Region**			0.001
	West	33 (32)	174 (51)	
	East	53 (52)	109 (32)	
	Other	16 (16)	60 (17)	
	**Country income**			0.010
	HIC	45 (44)	201 (57)	
	LMIC	57 (56)	142 (43)	
	**Surgeon experience**			0.450
	<50	21 (29)	70 (23)	
	50–199	20 (27)	103 (34)	
	≥200	32 (44)	127 (42)	
**Nurses (*n* = 239)**	**All**	104	135	
	**Region**			0.251
	West	22 (21)	40 (30)	
	East	57 (56)	61 (45)	
	Other	25 (24)	34 (25)	
	**Country income**			0.064
	HIC	24 (23)	46 (34)	
	LMIC	80 (77)	89 (66)	
	**Nurse experience (years)**			0.056
	0–5	59 (57)	59 (45)	
	>5	44 (43)	73 (55)	

Values in parentheses are percentages. HIC, high-income country; LMIC, low–middle-income country.

* χ^2^ test.

Consensus was reached to include 13 outcomes: disease-free survival, disease-specific survival, surgery-related death, recurrence of cancer, completeness of tumour removal, overall quality of life, nutritional effects, all-cause complications, intraoperative complications, anaesthetic complications, anastomotic complications, multiple organ failure, and bleeding.

Thirteen outcomes were categorized for exclusion, and no consensus was reached for 31 outcomes, which were subsequently discussed at the consensus meeting. An analysis exploring the relationship between participant characteristics (such as regional and demographic differences) and their impact on how outcomes were scored is under review^22^.

There was no statistically significant difference between the mean scores of participants completing both survey rounds and those completing round 1 only (mean(s.d.) difference 0.17(0.1), largest difference 0.4; *P* = 0.76).

### Consensus meeting

Forty-three Delphi survey participants (7 patients, 29 surgeons, 7 nurses) attended the consensus meeting in person (18) or using the online platform (25). Fourteen countries from four continents (South America, North America, Europe, Asia) were represented. A full breakdown of the regional origin of participants is described in *Appendix S1*. The difference in mean scores between consensus meeting participants and those completing round 2 of the survey was statistically significant (mean(s.d.) difference 0.3(0.23), largest difference 1.16; *P* < 0.001).

In preparation for the consensus meeting, the SMG reviewed and discussed the Delphi results. Of the 13 outcomes that reached consensus to be included, six related to perioperative complications. The SMG took the view that, as the outcome all-cause complications was voted for inclusion, by extension, all complications would need to be measured and reported by researchers as a minimum. However, 14 complication-type outcomes from the list of 57 did not reach consensus for inclusion and a further two outcomes reached consensus for exclusion from the COS. The SMG decided to present this seemingly contradictory position at the consensus meeting for further discussion and voting on a desired final position.

After an interactive debate, participants were asked to vote for one of five propositions: all complications to be reported individually as a minimum; all ‘serious’ (without defining the term serious) complications to be reported as a minimum; outcomes meeting the criteria as core as set out by the GASTROS study to be included; unsure; and other options. The result of this live vote was presented to participants, who were given an opportunity for further discussion ahead of a final vote. The result of the second vote is shown in *Appendix S1*. Votes were split between the first two options, with the lack of a clear consensus mandating the need for further work in this area. Consequently, all complication-type outcomes were excluded from further discussion.

Non-complication-type outcomes for which there was no consensus to include or exclude in the Delphi survey were then discussed. Results from the subsequent voting are presented in *Appendix S1*. No further outcomes from this no-consensus group were sufficiently prioritized for addition to the final COS. The final COS is listed in *Table 2*. Participants agreed that future work on complications, definitions, and when outcomes should be reported should involve both patients and healthcare professionals.

**Table 2:** Core outcome set for surgical trials in gastric cancer

	Core outcome
Surviving and controlling cancer	Disease-free survival
	Recurrence of cancer*
	Disease-specific survival
	Surgery-related death
	Completeness of tumour removal
Impact of surgery	Overall quality of life
	Nutritional effects
Complications	‘Serious’ adverse events^†^

* The outcome recurrence of cancer can be incorporated into the composite outcome disease-free survival. It is shown here as it separately reached consensus as a core outcome.

† No consensus was reached with respect to which outcomes should be reported as a minimum. ‘Serious’ adverse events should be reported as a minimum while further work is undertaken in this area.

### Protocol deviations

The original study protocol described a three-round Delphi survey. Based on emerging evidence at that time^23^, several COS developers have demonstrated that consensus can be reached with a two-round survey, which was less resource- and time-intensive. Consequently, the approach was altered and a two-round Delphi adopted survey in the present study.

## Discussion

The GASTROS study has developed the first COS for use in surgical trials for gastric cancer. This represents a significant step towards addressing the current challenges related to outcome reporting, synthesis of evidence, and research waste in this field. Outcomes in the set were identified as critically important through an inclusive international consensus process involving patients and healthcare professionals. Further work is required to develop the COS, in particular, finalizing definitions, seeking consensus on how complications should be included, and identifying appropriate outcome measurement instruments. In its present form, the COS will guide trialists to which outcome domains should be reported.

A COS can only achieve its stated aims if it is used by researchers. From the outset of the study, the SMG set out a clear strategy to ensure buy-in by researchers and professional bodies. This resulted in broad international support, and the development of a network that enabled the recruitment of over 1000 participants to the Delphi survey. These participants were well balanced in terms of regional origins and personal or professional experience of gastric cancer. A strength of the study is that the methodology used is based on consensus guidelines and has been transparently reported in detail at each stage^5^. Researchers can therefore be reassured that the COS has been through a robust development process and is a valid framework on which to base their research, regardless of where it takes place.

A COS is a minimum set of critically important outcomes. It does not limit trialists in their reporting of other outcomes of interest. Furthermore, it should be noted that some grant-awarding bodies can make their own recommendations regarding which outcomes should be reported as a minimum. An example would be the recommendation to report overall survival, which was not prioritized through the present consensus process^24^. As such, it is recommended that researchers ensure that additional outcomes selected in surgical trials for gastric cancer adequately reflect the opinions of patients and clinicians in their region, as well as taking into consideration other funding requirements. The authors also recommend that further work is done to examine the international applicability of the COS.

The study was unable to achieve agreement with respect to which complications should be included in the COS. The consensus meeting could not decide whether all complications or only ‘serious’ complications should be reported as a minimum. However, the overwhelming majority voted for one of these options, which will be the focus of future work in this area. Other surgical cancer-related COSs have differed in their recommendations for the reporting of complications. Some have included only a small number of ‘serious’ or ‘core’ complication-related outcomes^15^^,^^25^, whereas others have recommended the reporting of a broader collection of complications^26^. Based on discussions from the consensus meeting and the lack of agreement among other COS developers, the current recommendation is that all ‘serious’ adverse events should be reported as a minimum until this area is addressed further.

The term ‘serious’ was purposefully not defined at the consensus meeting so as not to remove focus from the discussions. Others have already attempted to define this; the Gastrectomy Complications Consensus Group (GCCG) is a collaboration of European surgeons who have prioritized a list of 27 clearly defined complications which should be reported as a minimum in research, audits, and registries^27^. They sought consensus through a Delphi process, although their methodology differs from that of the GASTROS study in that patients and non-European healthcare professionals did not participate. Currently, it is the only substantial work available in this field addressing the reporting of complications, and will undoubtedly contribute to, and shape, future work in this field, which is being developed by the first author.

Defining outcomes is an area that deserves further consideration. The present approach was to use plain-language descriptions to define outcomes presented in the Delphi survey and consensus meetings. These were developed with the support of the SAG and an independent patient group. This was necessary to ensure that patients were engaged throughout the study and made translations easier. It is acknowledged that these may not be adequately detailed for use in trials, and more work is required with researchers and patients to address this for the outcomes included in the COS. Substantial work has already been undertaken by the StEP-COMPAC (Standardized Endpoints in Perioperative Medicine—Core Outcome Measures in Perioperative and Anaesthetic Care) group to identify available definitions for outcomes from several systematic reviews^28–31^. As with the GCCG complication list, this process did not involve patients, which was contrary to the recommendation made by the GASTROS consensus meeting. Standardizing definitions for outcomes included in this COS will form part of stage 3 of the GASTROS study.

Identifying which outcomes to measure is the first step in standardizing outcome reporting in this field. Although many outcomes in the COS are event-type outcomes (such as complications, survival, and recurrence), some are composite outcomes which require the use of an instrument to measure (for example, quality of life and nutritional outcomes). There is currently no standardized approach to measuring these outcomes^32–34^, and selecting the best tools to measure them requires a robust methodological approach similar to the one employed in this study^35^^,^^36^. This will form the basis of future work.

There are limitations to the present study that require discussion. It could be argued that, given the multimodal nature of treatment for gastric cancer, the COS would be more relevant if it incorporated all therapies including chemotherapy, radiotherapy, and endotherapy. However, at the time that GASTROS was conceived, there were 24 ongoing surgical trials planning to recruit 11 000 patients for whom non-surgical-related outcomes would not be applicable or relevant. The timing of support from Japanese and Korean collaborators meant that their participation was through the English-language Delphi survey which likely influenced the recruitment of patients from these countries. Nonetheless, an exploratory analysis of the Delphi survey results suggested that patients from Eastern countries did not prioritize outcomes differently from their Western counterparts^22^. As discussed above, it is recommended that researchers work with patients as partners to determine additional outcomes that are important locally at the trial design phase.

Another consideration relates to the type of stakeholder groups recruited to the study. It was agreed to limit participation to patients, nurses, and surgeons as this represented a balance of a broad spectrum of opinion but ensured that the study’s coordination and data analysis were manageable. It should be acknowledged that other groups, such as caregivers, allied health professionals, regulators, policymakers, and grant awarding bodies, will also provide valuable opinion. Inclusion of these groups will be considered in future stages of the GASTROS study and when the COS is reviewed.

The consensus meeting was held in English, limiting participants to English speakers only. Although there was a broad spectrum of international representation from the surgeon group, this was not mirrored by the patient or nurse stakeholders who were primarily UK-based. That said, no further outcomes were added following discussions, supporting the validity of the Delphi process, which recruited widely in terms of regional origin and other demographic characteristics across all three stakeholder groups.

## Collaborators

GASTROS International Working Group Collaborators: S. Li; Y. L. He; Z. Xu; Y. Xue; H. Liang; G. Li; E. Zhao; P. Neumann; L. O'Neill; E. Guinan; D. Zanotti; G. de Manzoni; E. R. C. Hagens; M. I. van Berge Henegouwen; P. Lages; S. Onofre; R. M. Restrepo Nuñez; G. Salcedo Cabañas; M. Posada Gonzalez; C. Marin Campos; B. Candas; B. Emre Baki; M. Selim Bodur; R. Yildirim; A. Burak Cekic; J. Brown; K. Hayes. Participants in international Delphi survey: I. Daher; R. H. Gianchandani Moorjani; A. Adetoyese Adeyeye; A. Sulaiman Olayide; A. Mitsuo Leon-Takahashi; A. Pueyo Rabanal; A. Peri; A. Boddy; A. Novotny; A. Charalabopoulos; A. Alemdar; A. Souadka; A. M. Rodrigues Gomes; A. Lázaro; A. Maciel Da Silva; A. do Rosário da Conceição Silva e Santos; A. Guidi; A. J. Silva Bernardes; A. Quinn; A. Isik; A. A Slipek; B. Candaş Altinbaş; B. Johnson Alegbeleye; B. Wool Eom; B. Frittoli; B. Lonsdale; B. Rogers; B. J. Ammori; B. Rau; B. Molteni; B. E. Byrne; B. A. Villacís-Bermeo; B. E. Villacís Gallardo; B. Köse; C. J. Sampedro Nogueira; C. Loureiro; C. M. Oliveira de Sousa; C. G. Collins; C. Nonso Ekwunife; C. Chukwunwendu Osuagwu; C. L.-Y. Wong; C. Winkler; D. Reim; D. W. Kjær; D. Cooper; D. Horner; D. Irvine; D. J. Bowrey; D. J. Chuter; D. Elliot; D. McGhee; D. Toth; D. Öfner; D. K. Manatakis; D. R. Silveira Martins; E. J. T. Belt; E. Cattaneo; E. Samadov; E. Colak; E. Treppiedi; E. Guglielmi; E. Redondo-Villahoz; E. Ciferri; E. Tiemens-de Graaf; E. Cocozza; E. Pape; E. S. Drozdov; F. Enrico; F. Rashid; F. Marco; F. Rosa; F. Mingol Navarro; F. Simionato Perrotta; F. S.-Y. Chan; F. D. Saavedra Tomasich; F. R. Takeda; F. Farrell; F. Olanike Wuraola; G. Rosero; G. Bevilacqua; G. Baronio; G. Mura; G. de Manzoni; G. D'Eugenio; G. Ortega-Perez; G. Tilt; G. Sutcliffe; G. Mureddu; G. Guerra Jacob; G. H. Daneri; H. Olufemi Gbenga; H. Okabe; I. Kingsford Smith; I. Olawale Lateef; I. Garosio; İ. Hatipoğlu; I. Gockel; I. Negoi; I. S.-H. Min; I. M. M. Mesquita; I. Diez del Val; J. H. F. Leemhuis; J. A. Gossage; J. Weindelmayer; J. R. Izbicki; J. McKenzie Manson; J. Kelly; J. H. M. B. Stoot; J. W. Haveman; J. D. Brown; J. Sultan; J. Hassall; J. van Sandick; J. H. Saunders; J. K. Clarke; J. Heisterkamp; J. I. Vargas R; J. M. Couselo Villanueva; J. Ingmire; J. McEwen; J. Galindo Álvarez; J. Turner; J. Peng; K. Roberts; K. G. Brandon; K. Mitchell; K. McCarthy; K. Akhtar; K. N. Mikhailovich; L. Corbelli; L. Medeiros Milhomem; L. Solaini; L. Fengyuan; L. Xinchun; L. Timmermans; L. Porritt; L. Taglietti; L. Bonavina; L. F. Pinheiro; M. de los Angeles Mayo Ossorio; M. Schiavo; M. Marchesiello; M. das Dores Vieira Leite; M. DeMois; M. Posada Gonzalez; M. T. Di Felice; M. I. van Berge Henegouwen; M. D. de Sousa; M. Takahashi; M. Forshaw; M. Berselli; M. Paro; M. A. Usta; M.-H. Yan; M. Pinchin; M. CapriolI; M. Rubbini; M. Cowen; M. A. Herrera Servin; M.-Z. Li; M. Sasako; M. Shukri Jahit; M. Ngonyoku Muhinga; M. A. Tareen; M. F. Ahmad; M. S. Bodur; M. Kaban; N. Farooq; N. Coburn; N. Cooper; N. S. Blencowe; N. Loria; N. de Vries; N. Adami Andreollo; N. Köksal; N. Zanini; N. Kreuser; N. Okkabaz; O. Damiana; O. Afuwape; O. Kayode Fasiku; O. Comensoli; O. F. Koroye; P. Capener; P. Morgagni; P. M. Pernadas Lages; P. M. Wilkerson; P. Turner; P. Dutton; P. Hayes; P. Vorwald; P. Singh; Q. Gan; R. Kottayasamy Seenivasagam; R. Ayloor Seshadri; R. Guevara Castro; R. Douglas; R. M. Koshy; R. Yıldırım; R. J. E. Skipworth; R. A. Gould; R. C. Wetherill; R. Shaw; R. A. Burley; R. Palatucci; R. Racalbuto; R. M. Correia Casaca; S. M. Lagarde; S. Gana; S. Marietti; S. Qureshi; S. Morales-Conde; S. Molfino; S. G. Barreto; S. Turkyilmaz; S. Turan-Trabzon; S. Frisch; S. Castoldi; S. Belloni; S. Flisi; S. Galloway; S. R. Maria; S. Royston; T. Boyle; T. Ö. Sezer; V. Mengardo; V. Concepción Martín; V. Lee Wills; V. Owen-Holt; V. Casagrande; W. Al-Khyatt; W. Jansen; W. Wang; W. Eshuis; W. P. Polkowski; X. Huang; X. Wang; X.-Z. Chen; Y. Gonzalez Dominguez; Y. Wang; Y. K. S. Viswanath; Y.-L. He; Z. Demir; Z. Na.

## Acknowledgements

The authors highlight the role undertaken by A. Metryka, Senior Clinical Trials Coordinator, who facilitated the running of this study; and thank the following associations and groups for their support in facilitating recruitment to the GASTROS study Delphi survey: International Gastric Cancer Association (www.igca.info), Association of Upper Gastrointestinal Surgeons of Great Britain and Ireland (www.augis.org), Brazilian Gastric Cancer Association (www.abcg.org.br), Canadian Gastric Cancer Association (www.gastriccancer.ca), Chinese Gastric Cancer Association, Dutch Upper Gastrointestinal Cancer Group (www.ducg.nl), GASTRODATA group (www.gastrodata.org), Italian Research Group for Gastric Cancer (www.gircg.it), Korean Gastric Cancer Association (www.kgca-i.or.kr), Oesophago-Gastric Surgery Section of the Asociación Española de Cirujanos—Spain (www.aecirujanos.es), Upper Gastrointestinal International Robotic Association (www.ugira.org), United Kingdom Oncology Nursing Society (www.ukons.org.uk), European Oncology Nursing Society (www.cancernurse.eu), Oesophageal Patients Association—United Kingdom (www.opa.org.uk), My Gut Feeling—Canada (www.mygutfeeling.ca), No Stomach for Cancer—USA (www.nostomachforcancer.org), Vivere Senza Stomaco—Italy, Gastro/Oesophageal Support and Help Cancer Group (Bristol)—UK, and Greater Manchester Oesophago-Gastric Patient Support Group.

## Funding

This study was funded by a NIHR Doctoral Research Fellowship Grant (DRF-2015–08-023). J.M.B. is partially funded by the NIHR Bristol Biomedical Research Centre and the Medical Research Council (MRC) ConDUCT-II Hub for Trials Methodology Research. P.R.W. was funded by the MRC North West Hub for Trials Methodology Research (MR/K025635/01).

This paper presents independent research funded by the NIHR. The views expressed are those of the author(s) and not necessarily those of the NHS, the NIHR or the Department of Health.

## Data availability statement

P.R.W. and I.A.B. are joint senior authors. The data sets analysed for the present study are available from the corresponding author on reasonable request.

*Disclosure.* The authors declare no conflict of interest.

## Supplementary Material

znab192_Supplementary_Data
