# Supplementary material for: Core outcome set for surgical trials in gastric cancer (GASTROS study): international patient and healthcare professional consensus
Source: Br J Surg. 2021 Jun 24;108(10):1216–24. doi: 10.1093/bjs/znab192 (PMC10364901; doi:10.1093/bjs/znab192)
Supplement: znab192_Supplementary_Data [file znab192_supplementary_data.zip › Supplementary_file_1_-_COS-STAR_Check-list.docx]

### Supplementary file 1. Core Outcome Set-STandards for Reporting: The COS-STAR Statement.

| **SECTION/TOPIC** | **ITEM No.** | **CHECKLIST ITEM** | **Page** |
| --- | --- | --- | --- |
| **TITLE/ABSTRACT** |  |  |  |
| Title | 1a | Identify in the title that the paper reports the development of a COS |  |
| Abstract | 1b | Provide a structured summary |  |
| **INTRODUCTION** |  |  |  |
| Background and Objectives | 2a | Describe the background and explain the rationale for developing the COS. |  |
|  | 2b | Describe the specific objectives with reference to developing a COS. |  |
| Scope | 3a | Describe the health condition(s) and population(s) covered by the COS. |  |
|  | 3b | Describe the intervention(s) covered by the COS. |  |
|  | 3c | Describe the setting(s) in which the COS is to be applied. |  |
| **METHODS** |  |  |  |
| Protocol/Registry Entry | 4 | Indicate where the COS development protocol can be accessed, if available, and/or the study registration details. |  |
| Participants | 5 | Describe the rationale for stakeholder groups involved in the COS development process, eligibility criteria for participants from each group, and a description of how the individuals involved were identified. |  |
| Information Sources | 6a | Describe the information sources used to identify an initial list of outcomes. |  |
|  | 6b | Describe how outcomes were dropped/combined, with reasons (if applicable). |  |
| Consensus Process | 7 | Describe how the consensus process was undertaken. |  |
| Outcome Scoring | 8 | Describe how outcomes were scored and how scores were summarised. |  |
| Consensus Definition | 9a | Describe the consensus definition. |  |
|  | 9b | Describe the procedure for determining how outcomes were included or excluded from consideration during the consensus process. |  |
| Ethics and Consent | 10 | Provide a statement regarding the ethics and consent issues for the study. |  |
| **RESULTS** |  |  |  |
| Protocol Deviations | 11 | Describe any changes from the protocol (if applicable), with reasons, and describe what impact these changes have on the results. |  |
| Participants | 12 | Present data on the number and relevant characteristics of the people involved at all stages of COS development. |  |
| Outcomes | 13a | List all outcomes considered at the start of the consensus process. |  |
|  | 13b | Describe any new outcomes introduced and any outcomes dropped, with reasons, during the consensus process. |  |
| COS | 14 | List the outcomes in the final COS. |  |
| **DISCUSSION** |  |  |  |
| Limitations | 15 | Discuss any limitations in the COS development process. |  |
| Conclusions | 16 | Provide an interpretation of the final COS in the context of other evidence, and implications for future research. |  |
| **OTHER INFORMATION** |  |  |  |
| Funding | 17 | Describe sources of funding/role of funders. |  |
| Conflicts of Interest | 18 | Describe any conflicts of interest within the study team and how these were managed. |  |
