# Supplementary material for: Core outcome set for surgical trials in gastric cancer (GASTROS study): international patient and healthcare professional consensus
Source: Br J Surg. 2021 Jun 24;108(10):1216–24. doi: 10.1093/bjs/znab192 (PMC10364901; doi:10.1093/bjs/znab192)
Supplement: znab192_Supplementary_Data [file znab192_supplementary_data.zip › Supplementary_file_2_-_Rationalisation_process.docx]

### **Supplementary file 2. Rationalisation of outcomes from to original source by study management group. The ‘source’ column refers to where the outcome was identified: Interviews (qualitative interviews), Trials (systematic review of trials), PROs (domains from Patient-Reported Outcome measurement instruments).**

| **Source** | **Original Verbatim Outcome** | **Outcome** | **Outcome Area** | **Outcome Domain** |
| --- | --- | --- | --- | --- |
| Interviews | Anaesthetic Complications | Adverse drug reaction | Adverse events | Adverse events/effects |
| Interviews | Epidural Related Complications | Adverse drug reaction | Adverse events | Adverse events/effects |
| Interviews | Hallucinations | Adverse drug reaction | Adverse events | Adverse events/effects |
| Interviews | Medication-related complications | Adverse drug reaction | Adverse events | Adverse events/effects |
| Interviews | Medication-related complications | Adverse drug reaction | Adverse events | Adverse events/effects |
| Interviews | Bleeding | Organ, vascular and/or nerve injury | Adverse Events | Adverse events/effects |
| Interviews | Perforated bowel | Organ, vascular and/or nerve injury | Adverse Events | Adverse events/effects |
| Interviews | Cardiac Complications | Cardiac complications | Physiological/Clinical | Cardiac Outcomes |
| Interviews | Ability to have adjuvant chemotherapy | Ability to have adjuvant chemotherapy | Life Impact | Delivery of care |
| Interviews | Complete Excision of Cancer | Completeness of tumour resection | Life Impact | Delivery of care |
| Interviews | Excision of Lymph Nodes | Completeness of tumour resection | Life Impact | Delivery of care |
| Interviews | Need for splenectomy | Completeness of tumour resection | Life Impact | Delivery of care |
| Interviews | Operative time | Duration of surgery | Life Impact | Delivery of care |
| Interviews | Wound Size | Wound size | Life Impact | Delivery of care |
| Interviews | Body Image | Mental Health | Life Impact | Emotional functioning/wellbeing |
| Interviews | Insomnia | Mental Health | Life Impact | Emotional functioning/wellbeing |
| Interviews | Psychological impact | Mental Health | Life Impact | Emotional functioning/wellbeing |
| Interviews | Weight Loss | Mental Health | Life Impact | Emotional functioning/wellbeing |
| Interviews | Anastomotic Leak | Anastomotic complications | Physiological/Clinical | Gastrointestinal outcomes |
| Interviews | Anastomotic Stricture | Anastomotic complications | Physiological/Clinical | Gastrointestinal outcomes |
| Interviews | Belching | Gastrointestinal functional problems | Physiological/Clinical | Gastrointestinal outcomes |
| Interviews | Constipation | Gastrointestinal functional problems | Physiological/Clinical | Gastrointestinal outcomes |
| Interviews | Diarrhoea | Gastrointestinal functional problems | Physiological/Clinical | Gastrointestinal outcomes |
| Interviews | Dumping | Gastrointestinal functional problems | Physiological/Clinical | Gastrointestinal outcomes |
| Interviews | Gastrointestinal problems | Gastrointestinal functional problems | Physiological/Clinical | Gastrointestinal outcomes |
| Interviews | Gastrointestinal symptoms | Gastrointestinal functional problems | Physiological/Clinical | Gastrointestinal outcomes |
| Interviews | Nausea + Vomiting | Gastrointestinal functional problems | Physiological/Clinical | Gastrointestinal outcomes |
| Interviews | Reflux | Gastrointestinal functional problems | Physiological/Clinical | Gastrointestinal outcomes |
| Interviews | Adhesional | Other gastrointestinal complications | Physiological/Clinical | Gastrointestinal outcomes |
| Interviews | Intestinal complications | Other gastrointestinal complications | Physiological/Clinical | Gastrointestinal outcomes |
| Interviews | Small bowel obstruction | Other gastrointestinal complications | Physiological/Clinical | Gastrointestinal outcomes |
| Interviews | Time to start Eating and drinking | Return of Gastro-Intestinal function | Physiological/Clinical | Gastrointestinal outcomes |
| Interviews | Fatigue | Fatigue | Physiological/Clinical | General Outcomes |
| Interviews | Cramps | Pain | Physiological/Clinical | General Outcomes |
| Interviews | Long Term Pain | Pain | Physiological/Clinical | General Outcomes |
| Interviews | Pain | Pain | Physiological/Clinical | General Outcomes |
| Interviews | Post-op Pain | Pain | Physiological/Clinical | General Outcomes |
| Interviews | Overall QoL | Overall Quality of Life | Life Impact | Global Quality of Life |
| Interviews | Length of Stay Following Surgery | Duration of hospital stay | Resource Use | Hospital |
| Interviews | Re-Admission to Hospital | Readmission to hospital | Resource Use | Hospital |
| Interviews | B12 Deficiency | Nutritional complications | Physiological/Clinical | Metabolism and nutrition outcomes |
| Interviews | Eating & Drinking | Nutritional complications | Physiological/Clinical | Metabolism and nutrition outcomes |
| Interviews | Necessity of long-term feeding | Nutritional complications | Physiological/Clinical | Metabolism and nutrition outcomes |
| Interviews | Curing Cancer | Disease free survival | Death | Mortality/Survival |
| Interviews | Survival | Overall survival | Death | Mortality/Survival |
| Interviews | Post-operative Death | Surgery-related death | Death | Mortality/Survival |
| Interviews | Intra-operative Death | Surgery-related death | Death | Mortality/Survival |
| Interviews | Peri-operative death | Surgery-related death | Death | Mortality/Survival |
| Interviews | Hernia | Need for additional procedure | Resource Use | Need for intervention |
| Interviews | Need for future interventions | Need for additional procedure | Resource Use | Need for intervention |
| Interviews | Need for reintervention | Need for additional procedure | Resource Use | Need for intervention |
| Interviews | Re-Intervention | Need for additional procedure | Resource Use | Need for intervention |
| Interviews | Re-operation | Need for additional procedure | Resource Use | Need for intervention |
| Interviews | Recurrence of Cancer | Recurrence of cancer | Physiological/Clinical | Outcomes related to neoplasms |
| Interviews | Exercising | Social life and relationships | Life Impact | Physical Functioning |
| Interviews | Peripheral Oedema | Time to ambulation | Life Impact | Physical Functioning |
| Interviews | Post-op Mobility | Time to ambulation | Life Impact | Physical Functioning |
| Interviews | Catheter related complications | Urinary complications | Physiological/Clinical | Renal and urinary outcomes |
| Interviews | Pleural Effusion | Pulmonary complications | Physiological/Clinical | Respiratory, thoracic and mediastinal outcomes |
| Interviews | Pneumonia | Pulmonary complications | Physiological/Clinical | Respiratory, thoracic and mediastinal outcomes |
| Interviews | Pneumothorax | Pulmonary complications | Physiological/Clinical | Respiratory, thoracic and mediastinal outcomes |
| Interviews | Respiratory complications | Pulmonary complications | Physiological/Clinical | Respiratory, thoracic and mediastinal outcomes |
| Interviews | Normal Activities Affected | Activities of daily living and work/employment | Life Impact | Role functioning |
| Interviews | Returning to normal function | Activities of daily living and work/employment | Life Impact | Role functioning |
| Interviews | Returning to normality | Activities of daily living and work/employment | Life Impact | Role functioning |
| Interviews | Working | Activities of daily living and work/employment | Life Impact | Role functioning |
| Interviews | Wound Complications | Other Wound Complication | Physiological/Clinical | Skin and subcutaneous tissue outcomes |
| Interviews | Interacting with Others | Social life and relationships | Life Impact | Social functioning |
| Interviews | Reliance on Others | Social life and relationships | Life Impact | Social functioning |
| Interviews | Cerebro-vascular complications | Cerebrovascular complications | Physiological/Clinical | Vascular Outcomes |
| PROs | Problems with concentration and memory (cognitive function) | Cognitive Functioning | Life Impact | Cognitive Functioning |
| PROs | Spiritual or faith issues | Spiritual or faith issues | Life Impact | Cognitive Functioning |
| PROs | Problems with weight | Mental Health | Life Impact | Emotional functioning/wellbeing |
| PROs | Feeling in control of weight and appearance | Mental Health | Life Impact | Emotional functioning/wellbeing |
| PROs | Feeling satisfied/confident with one's body | Mental Health | Life Impact | Emotional functioning/wellbeing |
| PROs | Problems with anxiety | Mental health | Life Impact | Emotional functioning/wellbeing |
| PROs | Problems with depression | Mental health | Life Impact | Emotional functioning/wellbeing |
| PROs | Problems with changes in general mood | Mental health | Life Impact | Emotional functioning/wellbeing |
| PROs | Money worries due to loss of earnings (finances) | Mental health | Life Impact | Emotional functioning/wellbeing |
| PROs | Able to eat/drink more easily (dysphagia) | Gastrointestinal functional problems | Physiological/Clinical | Gastrointestinal outcomes |
| PROs | Able to swallow without pain (odynophagia) | Gastrointestinal functional problems | Physiological/Clinical | Gastrointestinal outcomes |
| PROs | Able to enjoy healthy/balanced eating pattern | Gastrointestinal functional problems | Physiological/Clinical | Gastrointestinal outcomes |
| PROs | Problems with acid indigestion/heartburn including at night (reflux) | Gastrointestinal functional problems | Physiological/Clinical | Gastrointestinal outcomes |
| PROs | Problems with regurgitation and/or vomiting | Gastrointestinal functional problems | Physiological/Clinical | Gastrointestinal outcomes |
| PROs | Belching, bloating or gas (flatulence) | Gastrointestinal functional problems | Physiological/Clinical | Gastrointestinal outcomes |
| PROs | Problems choking when eating/drinking | Gastrointestinal functional problems | Physiological/Clinical | Gastrointestinal outcomes |
| PROs | Problems with appetite loss | Gastrointestinal functional problems | Physiological/Clinical | Gastrointestinal outcomes |
| PROs | Problems with sense of taste | Gastrointestinal functional problems | Physiological/Clinical | Gastrointestinal outcomes |
| PROs | Sudden dizziness, sweating and/or feeling drained after eating (dumping) | Gastrointestinal functional problems | Physiological/Clinical | Gastrointestinal outcomes |
| PROs | Problems with feeling sick (nausea) | Gastrointestinal functional problems | Physiological/Clinical | Gastrointestinal outcomes |
| PROs | Problems with diarrhoea, including frequent bowel movements | Gastrointestinal functional problems | Physiological/Clinical | Gastrointestinal outcomes |
| PROs | Problems with weak voice/hoarseness | Gastrointestinal functional problems | Physiological/Clinical | Gastrointestinal outcomes |
| PROs | Problems with constipation | Gastrointestinal functional problems | Physiological/Clinical | Gastrointestinal outcomes |
| PROs | Problems with coughing | Gastrointestinal functional problems | Physiological/Clinical | Gastrointestinal outcomes |
| PROs | Problems with a dry mouth | Gastrointestinal functional problems | Physiological/Clinical | Gastrointestinal outcomes |
| PROs | Problems with tiredness (fatigue) | Fatigue | Physiological/Clinical | General Outcomes |
| PROs | Problems with general pain/discomfort | Pain | Physiological/Clinical | General Outcomes |
| PROs | Overall quality of life | Overall Quality of Life | Life Impact | Global Quality of Life |
| PROs | Having good general health | Physical health | Life Impact | Perceived health status |
| PROs | Able to carry out usual activities | Activities of daily living | Life Impact | Physical Functioning |
| PROs | Able to participate/enjoy physical activities | Activities of daily living | Life Impact | Physical Functioning |
| PROs | Problems with sleeping | Insomnia | Life Impact | Physical Functioning |
| PROs | Interested in and able to enjoy sex | Social life and relationships | Life Impact | Physical Functioning |
| PROs | Feeling out of breath/difficulties breathing (dyspnoea) | Pulmonary complications | Physiological/Clinical | Respiratory, thoracic and mediastinal outcomes |
| PROs | Problems with hair loss | Hair Loss | Physiological/Clinical | Skin and subcutaneous tissue outcomes |
| PROs | Problems eating socially | Ability to eat socially | Life Impact | Social Functioning |
| PROs | Able to have relationships with friends | Social life and relationships | Life Impact | Social Functioning |
| PROs | Able to have relationships with family members | Social life and relationships | Life Impact | Social Functioning |
| Trials | Adverse drug reaction | Adverse drug reaction | Adverse Events | Adverse events/effects |
| Trials | Complications number of | Any Complications | Adverse Events | Adverse events/effects |
| Trials | Early surgical complications | Any Complications | Adverse Events | Adverse events/effects |
| Trials | Hospital morbidity | Any Complications | Adverse Events | Adverse events/effects |
| Trials | Any complication | Any Complications | Adverse Events | Adverse events/effects |
| Trials | Complications | Any Complications | Adverse Events | Adverse events/effects |
| Trials | Morbidity | Any Complications | Adverse Events | Adverse events/effects |
| Trials | Morbidity rate | Any Complications | Adverse Events | Adverse events/effects |
| Trials | Number of patients with complications | Any Complications | Adverse Events | Adverse events/effects |
| Trials | Operative morbidity | Any Complications | Adverse Events | Adverse events/effects |
| Trials | Overall complications | Any Complications | Adverse Events | Adverse events/effects |
| Trials | Overall morbidity | Any Complications | Adverse Events | Adverse events/effects |
| Trials | Overall Post-operative complications | Any Complications | Adverse Events | Adverse events/effects |
| Trials | Peri-operative complications | Any Complications | Adverse Events | Adverse events/effects |
| Trials | Post-operative complications | Any Complications | Adverse Events | Adverse events/effects |
| Trials | Post-operative morbidity | Any Complications | Adverse Events | Adverse events/effects |
| Trials | post-operative surgical parameters | Any Complications | Adverse Events | Adverse events/effects |
| Trials | Post-operative symptoms | Any Complications | Adverse Events | Adverse events/effects |
| Trials | Procedure-related morbidity and mortality | Any Complications | Adverse Events | Adverse events/effects |
| Trials | Total complications | Any Complications | Adverse Events | Adverse events/effects |
| Trials | Total morbidity | Any Complications | Adverse Events | Adverse events/effects |
| Trials | Colonic perforation | Organ, vascular and/or nerve injury | Adverse Events | Adverse events/effects |
| Trials | Gastrointestinal injury | Organ, vascular and/or nerve injury | Adverse Events | Adverse events/effects |
| Trials | Iatrogenic spleen injury | Organ, vascular and/or nerve injury | Adverse Events | Adverse events/effects |
| Trials | idiopathic small bowel perforation | Organ, vascular and/or nerve injury | Adverse Events | Adverse events/effects |
| Trials | Pancreatic injury | Organ, vascular and/or nerve injury | Adverse Events | Adverse events/effects |
| Trials | Pancreatitis traumatic | Organ, vascular and/or nerve injury | Adverse Events | Adverse events/effects |
| Trials | Splenic injury | Organ, vascular and/or nerve injury | Adverse Events | Adverse events/effects |
| Trials | Thermal injury | Organ, vascular and/or nerve injury | Adverse Events | Adverse events/effects |
| Trials | Trocar related injury | Organ, vascular and/or nerve injury | Adverse Events | Adverse events/effects |
| Trials | Recurrent laryngeal nerve palsy | Organ, vascular and/or nerve injury | Adverse Events | Adverse events/effects |
| Trials | Splenic artery pseudoaneurysm | Organ, vascular and/or nerve injury | Adverse Events | Adverse events/effects |
| Trials | Allogenic blood transfusion | Organ, vascular and/or nerve injury | Adverse Events | Adverse events/effects |
| Trials | Amount of blood transfused | Organ, vascular and/or nerve injury | Adverse Events | Adverse events/effects |
| Trials | Bleeding | Organ, vascular and/or nerve injury | Adverse Events | Adverse events/effects |
| Trials | Bleeding abdominal | Organ, vascular and/or nerve injury | Adverse Events | Adverse events/effects |
| Trials | Blood transfusion | Organ, vascular and/or nerve injury | Adverse Events | Adverse events/effects |
| Trials | Blood transfusion volume | Organ, vascular and/or nerve injury | Adverse Events | Adverse events/effects |
| Trials | Gastrointestinal bleeding | Organ, vascular and/or nerve injury | Adverse Events | Adverse events/effects |
| Trials | Haemorrhage | Organ, vascular and/or nerve injury | Adverse Events | Adverse events/effects |
| Trials | Hb | Organ, vascular and/or nerve injury | Adverse Events | Adverse events/effects |
| Trials | intra-abdominal bleeding | Organ, vascular and/or nerve injury | Adverse Events | Adverse events/effects |
| Trials | Intraluminal bleeding | Organ, vascular and/or nerve injury | Adverse Events | Adverse events/effects |
| Trials | Intraoperative blood transfusion | Organ, vascular and/or nerve injury | Adverse Events | Adverse events/effects |
| Trials | intraoperative major bleeding | Organ, vascular and/or nerve injury | Adverse Events | Adverse events/effects |
| Trials | intraperitoneal haemorrhage | Organ, vascular and/or nerve injury | Adverse Events | Adverse events/effects |
| Trials | Need for blood transfusion | Organ, vascular and/or nerve injury | Adverse Events | Adverse events/effects |
| Trials | Peritoneal haemorrhage | Organ, vascular and/or nerve injury | Adverse Events | Adverse events/effects |
| Trials | Post-operative bleeding | Organ, vascular and/or nerve injury | Adverse Events | Adverse events/effects |
| Trials | Post-operative drain discharge | Organ, vascular and/or nerve injury | Adverse Events | Adverse events/effects |
| Trials | Post-operative hemorrhage | Organ, vascular and/or nerve injury | Adverse Events | Adverse events/effects |
| Trials | Transfusion | Organ, vascular and/or nerve injury | Adverse Events | Adverse events/effects |
| Trials | Transfusions received | Organ, vascular and/or nerve injury | Adverse Events | Adverse events/effects |
| Trials | Upper gastro-intestinal haemorrhage | Organ, vascular and/or nerve injury | Adverse Events | Adverse events/effects |
| Trials | Amount of bleeding | Organ, vascular and/or nerve injury | Adverse Events | Adverse events/effects |
| Trials | Amount of blood loss | Organ, vascular and/or nerve injury | Adverse Events | Adverse events/effects |
| Trials | Blood loss | Organ, vascular and/or nerve injury | Adverse Events | Adverse events/effects |
| Trials | Estimated blood loss | Organ, vascular and/or nerve injury | Adverse Events | Adverse events/effects |
| Trials | Intraoperative blood loss | Organ, vascular and/or nerve injury | Adverse Events | Adverse events/effects |
| Trials | Mean blood loss | Organ, vascular and/or nerve injury | Adverse Events | Adverse events/effects |
| Trials | Operative blood loss | Organ, vascular and/or nerve injury | Adverse Events | Adverse events/effects |
| Trials | Surgical complications | Surgical complications | Adverse Events | Adverse events/effects |
| Trials | Surgical risk | Surgical complications | Adverse Events | Adverse events/effects |
| Trials | Intraoperative complications | Surgical complications | Adverse Events | Adverse events/effects |
| Trials | Chyle leakage | Chyle leak | Physiological/Clinical | Blood and lymphatic system outcomes |
| Trials | Chylous drainage | Chyle leak | Physiological/Clinical | Blood and lymphatic system outcomes |
| Trials | Chylous leakage | Chyle leak | Physiological/Clinical | Blood and lymphatic system outcomes |
| Trials | Chylous lymphorrhea | Chyle leak | Physiological/Clinical | Blood and lymphatic system outcomes |
| Trials | Lymphatic leakage | Chyle leak | Physiological/Clinical | Blood and lymphatic system outcomes |
| Trials | Lymphorrhoea | Chyle leak | Physiological/Clinical | Blood and lymphatic system outcomes |
| Trials | Atrial fibrillation | Cardiac complications | Physiological/Clinical | Cardiac Outcomes |
| Trials | Cardiac complications | Cardiac complications | Physiological/Clinical | Cardiac Outcomes |
| Trials | Cardiac failure | Cardiac complications | Physiological/Clinical | Cardiac Outcomes |
| Trials | Cardiocirculatory | Cardiac complications | Physiological/Clinical | Cardiac Outcomes |
| Trials | Myocardial infarction | Cardiac complications | Physiological/Clinical | Cardiac Outcomes |
| Trials | R0 resection | Completeness of tumour resection | Life Impact | Delivery of Care |
| Trials | Radicality R0 | Completeness of tumour resection | Life Impact | Delivery of Care |
| Trials | Radicality R1 | Completeness of tumour resection | Life Impact | Delivery of Care |
| Trials | Residual Tumour | Completeness of tumour resection | Life Impact | Delivery of Care |
| Trials | Residual tumour R0 | Completeness of tumour resection | Life Impact | Delivery of Care |
| Trials | Residual tumour R1/2 | Completeness of tumour resection | Life Impact | Delivery of Care |
| Trials | Residual tumour R1/2 | Completeness of tumour resection | Life Impact | Delivery of Care |
| Trials | Clear margin distance | Completeness of tumour resection | Life Impact | Delivery of Care |
| Trials | Distal resection margin | Completeness of tumour resection | Life Impact | Delivery of Care |
| Trials | Proximal margin positive/negative | Completeness of tumour resection | Life Impact | Delivery of Care |
| Trials | Proximal resection margin | Completeness of tumour resection | Life Impact | Delivery of Care |
| Trials | Resection line involvement - distal | Completeness of tumour resection | Life Impact | Delivery of Care |
| Trials | Resection line involvement - proximal | Completeness of tumour resection | Life Impact | Delivery of Care |
| Trials | Length of lesser curvature of resected stomach | Completeness of tumour resection | Life Impact | Delivery of Care |
| Trials | Length of lesser curvature of resected stomach | Completeness of tumour resection | Life Impact | Delivery of Care |
| Trials | Length of resection on greater curve | Completeness of tumour resection | Life Impact | Delivery of Care |
| Trials | Length of resection on lesser curve | Completeness of tumour resection | Life Impact | Delivery of Care |
| Trials | Dissected Lymph nodes - mediastinal | Completeness of tumour resection | Life Impact | Delivery of Care |
| Trials | Dissected Lymph nodes - para-aortic | Completeness of tumour resection | Life Impact | Delivery of Care |
| Trials | Number of lymph nodes dissected or resected or retrieved | Completeness of tumour resection | Life Impact | Delivery of Care |
| Trials | Number of lymph nodes removed N1 group | Completeness of tumour resection | Life Impact | Delivery of Care |
| Trials | Number of lymph nodes removed N2 group | Completeness of tumour resection | Life Impact | Delivery of Care |
| Trials | Number of lymph nodes removed N3 group | Completeness of tumour resection | Life Impact | Delivery of Care |
| Trials | Number of lymph nodes removed N4 group | Completeness of tumour resection | Life Impact | Delivery of Care |
| Trials | Conversion to open surgery | Conversion to Open Surgery | Life Impact | Delivery of Care |
| Trials | Open conversion | Conversion to Open Surgery | Life Impact | Delivery of Care |
| Trials | Open conversion rate | Conversion to Open Surgery | Life Impact | Delivery of Care |
| Trials | Duration of surgery | Duration of Surgery | Life Impact | Delivery of Care |
| Trials | Mean operating time | Duration of Surgery | Life Impact | Delivery of Care |
| Trials | Operative time | Duration of Surgery | Life Impact | Delivery of Care |
| Trials | Surgical time | Duration of Surgery | Life Impact | Delivery of Care |
| Trials | Time for operation | Duration of Surgery | Life Impact | Delivery of Care |
| Trials | length of incision | Wound size | Life Impact | Delivery of Care |
| Trials | Length of laparotomy incision | Wound size | Life Impact | Delivery of Care |
| Trials | Length of longest wound | Wound size | Life Impact | Delivery of Care |
| Trials | Main wound size (cm) | Wound size | Life Impact | Delivery of Care |
| Trials | Size of wound | Wound size | Life Impact | Delivery of Care |
| Trials | Total length (of wound) | Wound size | Life Impact | Delivery of Care |
| Trials | Wound length (cm) | Wound size | Life Impact | Delivery of Care |
| Trials | Medical cost | Cost | Resource Use | Economic |
| Trials | Post-operative glucose tolerance | Endocrine complications | Physiological/Clinical | Endocrine outcomes |
| Trials | endocrine complications | Endocrine complications | Physiological/Clinical | Endocrine outcomes |
| Trials | endocrine events | Endocrine complications | Physiological/Clinical | Endocrine outcomes |
| Trials | Metabolic complications | Endocrine complications | Physiological/Clinical | Endocrine outcomes |
| Trials | Anastomosis failure | Anastomotic complications | Physiological/Clinical | Gastrointestinal outcomes |
| Trials | Anastomotic dehiscence | Anastomotic complications | Physiological/Clinical | Gastrointestinal outcomes |
| Trials | Anastomotic leak | Anastomotic complications | Physiological/Clinical | Gastrointestinal outcomes |
| Trials | Anastomotic leakage from GJ | Anastomotic complications | Physiological/Clinical | Gastrointestinal outcomes |
| Trials | Anastomotic leakage from OJ | Anastomotic complications | Physiological/Clinical | Gastrointestinal outcomes |
| Trials | Anastomotic leakage type 1 | Anastomotic complications | Physiological/Clinical | Gastrointestinal outcomes |
| Trials | Anastomotic leakage type 2 | Anastomotic complications | Physiological/Clinical | Gastrointestinal outcomes |
| Trials | Leakage | Anastomotic complications | Physiological/Clinical | Gastrointestinal outcomes |
| Trials | Minor leakage | Anastomotic complications | Physiological/Clinical | Gastrointestinal outcomes |
| Trials | Esophagus and remnant stomach infarction | Anastomotic complications | Physiological/Clinical | Gastrointestinal outcomes |
| Trials | Gastric remnant necrosis | Anastomotic complications | Physiological/Clinical | Gastrointestinal outcomes |
| Trials | Duodenal leak | Anastomotic complications | Physiological/Clinical | Gastrointestinal outcomes |
| Trials | Duodenal stump leak | Anastomotic complications | Physiological/Clinical | Gastrointestinal outcomes |
| Trials | Duodenal stump leakage | Anastomotic complications | Physiological/Clinical | Gastrointestinal outcomes |
| Trials | Anastomotic bleeding | Anastomotic complications | Physiological/Clinical | Gastrointestinal outcomes |
| Trials | Bleeding from anastomosis | Anastomotic complications | Physiological/Clinical | Gastrointestinal outcomes |
| Trials | Anastomosis stricture | Anastomotic complications | Physiological/Clinical | Gastrointestinal outcomes |
| Trials | Anastomotic stenosis | Anastomotic complications | Physiological/Clinical | Gastrointestinal outcomes |
| Trials | Stenosis | Anastomotic complications | Physiological/Clinical | Gastrointestinal outcomes |
| Trials | Delayed gastric emptying | Gastrointestinal functional problems | Physiological/Clinical | Gastrointestinal outcomes |
| Trials | Delayed gastric emptying without obstruction | Gastrointestinal functional problems | Physiological/Clinical | Gastrointestinal outcomes |
| Trials | Gastric atonia | Gastrointestinal functional problems | Physiological/Clinical | Gastrointestinal outcomes |
| Trials | Gastroparesis | Gastrointestinal functional problems | Physiological/Clinical | Gastrointestinal outcomes |
| Trials | Stasis | Gastrointestinal functional problems | Physiological/Clinical | Gastrointestinal outcomes |
| Trials | Rate of reinsertion of NG tube | Gastrointestinal functional problems | Physiological/Clinical | Gastrointestinal outcomes |
| Trials | Diarrhoea | Gastrointestinal functional problems | Physiological/Clinical | Gastrointestinal outcomes |
| Trials | Prolonged diarrhea | Gastrointestinal functional problems | Physiological/Clinical | Gastrointestinal outcomes |
| Trials | Severe diarrhoea | Gastrointestinal functional problems | Physiological/Clinical | Gastrointestinal outcomes |
| Trials | Dumping syndrome | Gastrointestinal functional problems | Physiological/Clinical | Gastrointestinal outcomes |
| Trials | Early dumping syndrome | Gastrointestinal functional problems | Physiological/Clinical | Gastrointestinal outcomes |
| Trials | Hiccups | Gastrointestinal functional problems | Physiological/Clinical | Gastrointestinal outcomes |
| Trials | Nausea | Gastrointestinal functional problems | Physiological/Clinical | Gastrointestinal outcomes |
| Trials | Reflux oesophagitis | Gastrointestinal functional problems | Physiological/Clinical | Gastrointestinal outcomes |
| Trials | Vomiting | Gastrointestinal functional problems | Physiological/Clinical | Gastrointestinal outcomes |
| Trials | Malabsorption | Nutritional complications | Physiological/Clinical | Gastrointestinal outcomes |
| Trials | Severe feeding problem requiring prolonged hyperalimentation | Nutritional complications | Physiological/Clinical | Gastrointestinal outcomes |
| Trials | Abdominal distention | Other gastrointestinal complications | Physiological/Clinical | Gastrointestinal outcomes |
| Trials | Acute enteritis | Other gastrointestinal complications | Physiological/Clinical | Gastrointestinal outcomes |
| Trials | Gastrointestinal complications | Other gastrointestinal complications | Physiological/Clinical | Gastrointestinal outcomes |
| Trials | Enterocutaneous fistula | Other gastrointestinal complications | Physiological/Clinical | Gastrointestinal outcomes |
| Trials | Enterocutaneous fistulas | Other gastrointestinal complications | Physiological/Clinical | Gastrointestinal outcomes |
| Trials | Intestinal fistula | Other gastrointestinal complications | Physiological/Clinical | Gastrointestinal outcomes |
| Trials | Intestinal ischaemia | Other gastrointestinal complications | Physiological/Clinical | Gastrointestinal outcomes |
| Trials | Afferent loop syndrome | Other gastrointestinal complications | Physiological/Clinical | Gastrointestinal outcomes |
| Trials | A-loop syndrome | Other gastrointestinal complications | Physiological/Clinical | Gastrointestinal outcomes |
| Trials | Bowel obstruction | Other gastrointestinal complications | Physiological/Clinical | Gastrointestinal outcomes |
| Trials | Bowel obstruction/ileus | Other gastrointestinal complications | Physiological/Clinical | Gastrointestinal outcomes |
| Trials | Ileus mechanical | Other gastrointestinal complications | Physiological/Clinical | Gastrointestinal outcomes |
| Trials | Ileus adhesive | Other gastrointestinal complications | Physiological/Clinical | Gastrointestinal outcomes |
| Trials | intestinal obstruction | Other gastrointestinal complications | Physiological/Clinical | Gastrointestinal outcomes |
| Trials | Small-bowel obstruction | Other gastrointestinal complications | Physiological/Clinical | Gastrointestinal outcomes |
| Trials | Ileus | Other gastrointestinal complications | Physiological/Clinical | Gastrointestinal outcomes |
| Trials | Ileus paralytic | Other gastrointestinal complications | Physiological/Clinical | Gastrointestinal outcomes |
| Trials | Ileus prolonged | Other gastrointestinal complications | Physiological/Clinical | Gastrointestinal outcomes |
| Trials | Days till first flatus | Return of Gastro-Intestinal function | Physiological/Clinical | Gastrointestinal outcomes |
| Trials | Days to first flatus | Return of Gastro-Intestinal function | Physiological/Clinical | Gastrointestinal outcomes |
| Trials | Days to sips of water | Return of Gastro-Intestinal function | Physiological/Clinical | Gastrointestinal outcomes |
| Trials | Eating | Return of Gastro-Intestinal function | Physiological/Clinical | Gastrointestinal outcomes |
| Trials | First eating (post-operative day) | Return of Gastro-Intestinal function | Physiological/Clinical | Gastrointestinal outcomes |
| Trials | First flatus | Return of Gastro-Intestinal function | Physiological/Clinical | Gastrointestinal outcomes |
| Trials | First flatus (post-operative day) | Return of Gastro-Intestinal function | Physiological/Clinical | Gastrointestinal outcomes |
| Trials | Food intake | Return of Gastro-Intestinal function | Physiological/Clinical | Gastrointestinal outcomes |
| Trials | Progression of oral intake | Return of Gastro-Intestinal function | Physiological/Clinical | Gastrointestinal outcomes |
| Trials | Time of first flatus/index of peristalsis recovery | Return of Gastro-Intestinal function | Physiological/Clinical | Gastrointestinal outcomes |
| Trials | Time to first flatus | Return of Gastro-Intestinal function | Physiological/Clinical | Gastrointestinal outcomes |
| Trials | Time to first flatus (days) | Return of Gastro-Intestinal function | Physiological/Clinical | Gastrointestinal outcomes |
| Trials | Time to first liquid intake | Return of Gastro-Intestinal function | Physiological/Clinical | Gastrointestinal outcomes |
| Trials | Time to first soft diet uptake | Return of Gastro-Intestinal function | Physiological/Clinical | Gastrointestinal outcomes |
| Trials | Time to flatus (postoperative days) | Return of Gastro-Intestinal function | Physiological/Clinical | Gastrointestinal outcomes |
| Trials | Time to food intake | Return of Gastro-Intestinal function | Physiological/Clinical | Gastrointestinal outcomes |
| Trials | Time to liquid diet | Return of Gastro-Intestinal function | Physiological/Clinical | Gastrointestinal outcomes |
| Trials | Time to sips of water | Return of Gastro-Intestinal function | Physiological/Clinical | Gastrointestinal outcomes |
| Trials | Time to start oral intake (days) | Return of Gastro-Intestinal function | Physiological/Clinical | Gastrointestinal outcomes |
| Trials | Time until removal of the naso-gastric tube | Return of Gastro-Intestinal function | Physiological/Clinical | Gastrointestinal outcomes |
| Trials | Time until start of meals | Return of Gastro-Intestinal function | Physiological/Clinical | Gastrointestinal outcomes |
| Trials | Multiple organ failure | Multiple organ failure | Physiological/Clinical | General Outcomes |
| Trials | Body weight (kg) | Nutritional status | Physiological/Clinical | General Outcomes |
| Trials | Decrease in body weight | Nutritional status | Physiological/Clinical | General Outcomes |
| Trials | Decrease of body weight 1 month after the operation | Nutritional status | Physiological/Clinical | General Outcomes |
| Trials | Lean body mass | Nutritional status | Physiological/Clinical | General Outcomes |
| Trials | Nutritional Status | Nutritional status | Physiological/Clinical | General Outcomes |
| Trials | Prealbumin (gm/dL) | Nutritional status | Physiological/Clinical | General Outcomes |
| Trials | Serum Albumin | Nutritional status | Physiological/Clinical | General Outcomes |
| Trials | Total body weight | Nutritional status | Physiological/Clinical | General Outcomes |
| Trials | Total protein | Nutritional status | Physiological/Clinical | General Outcomes |
| Trials | Degree of pain | Pain | Physiological/Clinical | General Outcomes |
| Trials | Pain | Pain | Physiological/Clinical | General Outcomes |
| Trials | Post-operative Pain | Pain | Physiological/Clinical | General Outcomes |
| Trials | Post-operative pain | Pain | Physiological/Clinical | General Outcomes |
| Trials | Residual pain at day 7 | Pain | Physiological/Clinical | General Outcomes |
| Trials | Body temperature exceeding 37 ° C (days) | Surgical stress response | Physiological/Clinical | General Outcomes |
| Trials | Fever | Surgical stress response | Physiological/Clinical | General Outcomes |
| Trials | CK | Surgical stress response | Physiological/Clinical | General Outcomes |
| Trials | CRP | Surgical stress response | Physiological/Clinical | General Outcomes |
| Trials | CRP 3 days after surgery | Surgical stress response | Physiological/Clinical | General Outcomes |
| Trials | Days of fever | Surgical stress response | Physiological/Clinical | General Outcomes |
| Trials | Fever | Surgical stress response | Physiological/Clinical | General Outcomes |
| Trials | IL-6 | Surgical stress response | Physiological/Clinical | General Outcomes |
| Trials | Immediate postoperative inflammatory and immune responses | Surgical stress response | Physiological/Clinical | General Outcomes |
| Trials | Immunological response after surgery | Surgical stress response | Physiological/Clinical | General Outcomes |
| Trials | Immunological response to surgery | Surgical stress response | Physiological/Clinical | General Outcomes |
| Trials | Surgical stress response | Surgical stress response | Physiological/Clinical | General Outcomes |
| Trials | WBC (/mm3) | Surgical stress response | Physiological/Clinical | General Outcomes |
| Trials | WCC | Surgical stress response | Physiological/Clinical | General Outcomes |
| Trials | QoL | Overall Quality of Life | Life Impact | Global Quality of Life |
| Trials | Overall satisfaction | Overall Quality of Life | Life Impact | Global Quality of Life |
| Trials | Cholecystitis | Gallbladder-related complications | Physiological/Clinical | Hepatobiliary Outcomes |
| Trials | Cholecystitis acute | Gallbladder-related complications | Physiological/Clinical | Hepatobiliary Outcomes |
| Trials | Cholecystitis requiring percutaneous drainage | Gallbladder-related complications | Physiological/Clinical | Hepatobiliary Outcomes |
| Trials | Presence of gallstones | Gallbladder-related complications | Physiological/Clinical | Hepatobiliary Outcomes |
| Trials | Drug-induced hepatitis | Hepatic complications | Physiological/Clinical | Hepatobiliary Outcomes |
| Trials | Hepatic complications | Hepatic complications | Physiological/Clinical | Hepatobiliary Outcomes |
| Trials | Hepatic failure | Hepatic complications | Physiological/Clinical | Hepatobiliary Outcomes |
| Trials | Liver dysfunction | Hepatic complications | Physiological/Clinical | Hepatobiliary Outcomes |
| Trials | Transient LFT abnormality | Hepatic complications | Physiological/Clinical | Hepatobiliary Outcomes |
| Trials | Blood urea nitrogen | Hepatic complications | Physiological/Clinical | Hepatobiliary Outcomes |
| Trials | LFT | Hepatic complications | Physiological/Clinical | Hepatobiliary Outcomes |
| Trials | Pancreatitis | Pancreas-related complications | Physiological/Clinical | Hepatobiliary Outcomes |
| Trials | Pancreatitis acute | Pancreas-related complications | Physiological/Clinical | Hepatobiliary Outcomes |
| Trials | Pancreatitis edematous | Pancreas-related complications | Physiological/Clinical | Hepatobiliary Outcomes |
| Trials | Pancreatitis severe | Pancreas-related complications | Physiological/Clinical | Hepatobiliary Outcomes |
| Trials | Pancreas-related complications | Pancreas-related complications | Physiological/Clinical | Hepatobiliary Outcomes |
| Trials | Abdominal drainage | Pancreas-related complications | Physiological/Clinical | Hepatobiliary Outcomes |
| Trials | Amylase level in drainage fluid | Pancreas-related complications | Physiological/Clinical | Hepatobiliary Outcomes |
| Trials | Minor discharge of pancreatic juice | Pancreas-related complications | Physiological/Clinical | Hepatobiliary Outcomes |
| Trials | Pancreatic fistula | Pancreas-related complications | Physiological/Clinical | Hepatobiliary Outcomes |
| Trials | Pancreatic leak | Pancreas-related complications | Physiological/Clinical | Hepatobiliary Outcomes |
| Trials | Pancreatic leakage | Pancreas-related complications | Physiological/Clinical | Hepatobiliary Outcomes |
| Trials | Days of hospitalization | Duration of hospital stay | Resource Use | Hospital |
| Trials | Duration of hospital stay | Duration of hospital stay | Resource Use | Hospital |
| Trials | Duration of post-operative hospital stay | Duration of hospital stay | Resource Use | Hospital |
| Trials | Readmission | Readmission to hospital | Resource Use | Hospital |
| Trials | Abdominal abscess | Abdominal collection | Physiological/Clinical | Infection and infestation outcomes |
| Trials | Abdominal liquid accumulation | Abdominal collection | Physiological/Clinical | Infection and infestation outcomes |
| Trials | Abscess intra-abdominal | Abdominal collection | Physiological/Clinical | Infection and infestation outcomes |
| Trials | Abscess subphrenic | Abdominal collection | Physiological/Clinical | Infection and infestation outcomes |
| Trials | Abscesses Intra-abdominal | Abdominal collection | Physiological/Clinical | Infection and infestation outcomes |
| Trials | Ascites | Abdominal collection | Physiological/Clinical | Infection and infestation outcomes |
| Trials | Fluid collection | Abdominal collection | Physiological/Clinical | Infection and infestation outcomes |
| Trials | Fluid collection/abscesses | Abdominal collection | Physiological/Clinical | Infection and infestation outcomes |
| Trials | Intra-abdominal collections | Abdominal collection | Physiological/Clinical | Infection and infestation outcomes |
| Trials | intra-abdominal infection | Abdominal collection | Physiological/Clinical | Infection and infestation outcomes |
| Trials | Major abdominal infections | Abdominal collection | Physiological/Clinical | Infection and infestation outcomes |
| Trials | Prolonged retention of intra-abdominal fluid | Abdominal collection | Physiological/Clinical | Infection and infestation outcomes |
| Trials | Infection | Other Infection | Physiological/Clinical | Infection and infestation outcomes |
| Trials | Herpes zoster | Other Infection | Physiological/Clinical | Infection and infestation outcomes |
| Trials | Viral infection | Other Infection | Physiological/Clinical | Infection and infestation outcomes |
| Trials | Mediastinitis | Other Infection | Physiological/Clinical | Infection and infestation outcomes |
| Trials | Septic complications | Other Infection | Physiological/Clinical | Infection and infestation outcomes |
| Trials | Systemic infections | Other Infection | Physiological/Clinical | Infection and infestation outcomes |
| Trials | Disease free survival | Disease free survival | Death | Mortality/Survival |
| Trials | Disease free survival 4-year | Disease free survival | Death | Mortality/Survival |
| Trials | Disease free survival 5-year | Disease free survival | Death | Mortality/Survival |
| Trials | Recurrence-free survival | Disease free survival | Death | Mortality/Survival |
| Trials | Relapse-free survival | Disease free survival | Death | Mortality/Survival |
| Trials | Death from gastric cancer as a cause | Disease specific survival | Death | Mortality/Survival |
| Trials | Disease specific survival | Disease specific survival | Death | Mortality/Survival |
| Trials | Disease specific survival 5-year | Disease specific survival | Death | Mortality/Survival |
| Trials | Gastric cancer related deaths | Disease specific survival | Death | Mortality/Survival |
| Trials | Overall survival | Overall survival | Death | Mortality/Survival |
| Trials | Overall survival 10-year | Overall survival | Death | Mortality/Survival |
| Trials | Overall survival 3-year | Overall survival | Death | Mortality/Survival |
| Trials | Overall survival 5-year | Overall survival | Death | Mortality/Survival |
| Trials | Overall survival 6-year | Overall survival | Death | Mortality/Survival |
| Trials | Overall survival 7-year | Overall survival | Death | Mortality/Survival |
| Trials | Survival 11-year | Overall survival | Death | Mortality/Survival |
| Trials | Survival 5-year | Overall survival | Death | Mortality/Survival |
| Trials | Survival Period | Overall survival | Death | Mortality/Survival |
| Trials | Death | Surgery-related death | Death | Mortality/Survival |
| Trials | Death from a post-operative complication | Surgery-related death | Death | Mortality/Survival |
| Trials | Death from all causes | Surgery-related death | Death | Mortality/Survival |
| Trials | Hospital death | Surgery-related death | Death | Mortality/Survival |
| Trials | Hospital mortality | Surgery-related death | Death | Mortality/Survival |
| Trials | In-hospital mortality | Surgery-related death | Death | Mortality/Survival |
| Trials | Mortality | Surgery-related death | Death | Mortality/Survival |
| Trials | Mortality from all causes | Surgery-related death | Death | Mortality/Survival |
| Trials | Mortality not related to surgery | Surgery-related death | Death | Mortality/Survival |
| Trials | Operative death | Surgery-related death | Death | Mortality/Survival |
| Trials | Operative mortality | Surgery-related death | Death | Mortality/Survival |
| Trials | Post-operative death | Surgery-related death | Death | Mortality/Survival |
| Trials | Post-operative mortality | Surgery-related death | Death | Mortality/Survival |
| Trials | Post-operative survival | Surgery-related death | Death | Mortality/Survival |
| Trials | Treatment related deaths | Surgery-related death | Death | Mortality/Survival |
| Trials | Re-laparotomy | Need for additional procedure | Resource Use | Need for intervention |
| Trials | Re-operation | Need for additional procedure | Resource Use | Need for intervention |
| Trials | Re-operation details | Need for additional procedure | Resource Use | Need for intervention |
| Trials | Return to theatre | Need for additional procedure | Resource Use | Need for intervention |
| Trials | 4-day post-operative use of analgesics | Need for analgesia | Resource Use | Need for intervention |
| Trials | Dose of analgesic (mg) | Need for analgesia | Resource Use | Need for intervention |
| Trials | Duration of pain control | Need for analgesia | Resource Use | Need for intervention |
| Trials | Frequency of analgesics injection | Need for analgesia | Resource Use | Need for intervention |
| Trials | Frequency of injection given according to analgesic requests | Need for analgesia | Resource Use | Need for intervention |
| Trials | Pain control | Need for analgesia | Resource Use | Need for intervention |
| Trials | Post-operative analgesia | Need for analgesia | Resource Use | Need for intervention |
| Trials | Time to removal of epidural anesthesia (days) | Need for analgesia | Resource Use | Need for intervention |
| Trials | Times analgesic given | Need for analgesia | Resource Use | Need for intervention |
| Trials | Times of pain rescue | Need for analgesia | Resource Use | Need for intervention |
| Trials | Total amount of analgesics infused (mL) | Need for analgesia | Resource Use | Need for intervention |
| Trials | Cumulative risk of recurrence | Recurrence of cancer | Physiological/Clinical | Outcomes related to neoplasms |
| Trials | Cumulative risk of relapse | Recurrence of cancer | Physiological/Clinical | Outcomes related to neoplasms |
| Trials | Disease recurrence rate | Recurrence of cancer | Physiological/Clinical | Outcomes related to neoplasms |
| Trials | port site metastasis | Recurrence of cancer | Physiological/Clinical | Outcomes related to neoplasms |
| Trials | Recurrence | Recurrence of cancer | Physiological/Clinical | Outcomes related to neoplasms |
| Trials | Recurrence patterns | Recurrence of cancer | Physiological/Clinical | Outcomes related to neoplasms |
| Trials | Recurrent disease | Recurrence of cancer | Physiological/Clinical | Outcomes related to neoplasms |
| Trials | Tumor recurrence | Recurrence of cancer | Physiological/Clinical | Outcomes related to neoplasms |
| Trials | First walking (post-operative day) | Time to ambulation | Life Impact | Physical functioning |
| Trials | Number of days to get out of bed | Time to ambulation | Life Impact | Physical functioning |
| Trials | Recovery of Physical Activity | Time to ambulation | Life Impact | Physical functioning |
| Trials | Time to ambulation | Time to ambulation | Life Impact | Physical functioning |
| Trials | Walking | Time to ambulation | Life Impact | Physical functioning |
| Trials | Post-operative psychosis | Post-operative psychosis | Physiological/Clinical | Psychiatric Outcomes |
| Trials | Renal complications | Renal complications | Physiological/Clinical | Renal and urinary outcomes |
| Trials | Renal failure | Renal complications | Physiological/Clinical | Renal and urinary outcomes |
| Trials | Acute urinary retention | Urinary complications | Physiological/Clinical | Renal and urinary outcomes |
| Trials | Catheter-induced sepsis | Urinary complications | Physiological/Clinical | Renal and urinary outcomes |
| Trials | Urinary complications | Urinary complications | Physiological/Clinical | Renal and urinary outcomes |
| Trials | Urinary retention | Urinary complications | Physiological/Clinical | Renal and urinary outcomes |
| Trials | Urinary tract complications | Urinary complications | Physiological/Clinical | Renal and urinary outcomes |
| Trials | Urinary tract infection | Urinary complications | Physiological/Clinical | Renal and urinary outcomes |
| Trials | Hypercapnia | Pulmonary complications | Physiological/Clinical | Respiratory, thoracic and mediastinal outcomes |
| Trials | Atelectasis | Pulmonary complications | Physiological/Clinical | Respiratory, thoracic and mediastinal outcomes |
| Trials | Atelectasis or pleural effusion | Pulmonary complications | Physiological/Clinical | Respiratory, thoracic and mediastinal outcomes |
| Trials | Bronchopneumonia | Pulmonary complications | Physiological/Clinical | Respiratory, thoracic and mediastinal outcomes |
| Trials | Bronchoscopic toilet | Pulmonary complications | Physiological/Clinical | Respiratory, thoracic and mediastinal outcomes |
| Trials | Cardiopulmonary disease | Pulmonary complications | Physiological/Clinical | Respiratory, thoracic and mediastinal outcomes |
| Trials | empyema thoracis | Pulmonary complications | Physiological/Clinical | Respiratory, thoracic and mediastinal outcomes |
| Trials | Lung Infection | Pulmonary complications | Physiological/Clinical | Respiratory, thoracic and mediastinal outcomes |
| Trials | Major cardiorespiratory incidents | Pulmonary complications | Physiological/Clinical | Respiratory, thoracic and mediastinal outcomes |
| Trials | Minor patchy pulmonary collapse | Pulmonary complications | Physiological/Clinical | Respiratory, thoracic and mediastinal outcomes |
| Trials | Minor pulmonary atelectasis | Pulmonary complications | Physiological/Clinical | Respiratory, thoracic and mediastinal outcomes |
| Trials | Pleural | Pulmonary complications | Physiological/Clinical | Respiratory, thoracic and mediastinal outcomes |
| Trials | Pleural effusion | Pulmonary complications | Physiological/Clinical | Respiratory, thoracic and mediastinal outcomes |
| Trials | Pleural fluid | Pulmonary complications | Physiological/Clinical | Respiratory, thoracic and mediastinal outcomes |
| Trials | Pneumonia | Pulmonary complications | Physiological/Clinical | Respiratory, thoracic and mediastinal outcomes |
| Trials | Post-operative respiratory care | Pulmonary complications | Physiological/Clinical | Respiratory, thoracic and mediastinal outcomes |
| Trials | Post-operative respiratory function | Pulmonary complications | Physiological/Clinical | Respiratory, thoracic and mediastinal outcomes |
| Trials | Pulmonary | Pulmonary complications | Physiological/Clinical | Respiratory, thoracic and mediastinal outcomes |
| Trials | Pulmonary complications | Pulmonary complications | Physiological/Clinical | Respiratory, thoracic and mediastinal outcomes |
| Trials | Pulmonary edema | Pulmonary complications | Physiological/Clinical | Respiratory, thoracic and mediastinal outcomes |
| Trials | Pulmonary infection | Pulmonary complications | Physiological/Clinical | Respiratory, thoracic and mediastinal outcomes |
| Trials | Pyothorax | Pulmonary complications | Physiological/Clinical | Respiratory, thoracic and mediastinal outcomes |
| Trials | Respirator use after surgery | Pulmonary complications | Physiological/Clinical | Respiratory, thoracic and mediastinal outcomes |
| Trials | Respiratory complications | Pulmonary complications | Physiological/Clinical | Respiratory, thoracic and mediastinal outcomes |
| Trials | Respiratory failure | Pulmonary complications | Physiological/Clinical | Respiratory, thoracic and mediastinal outcomes |
| Trials | Thoracic effusion requiring thoracic drainage | Pulmonary complications | Physiological/Clinical | Respiratory, thoracic and mediastinal outcomes |
| Trials | Tracheotomy | Pulmonary complications | Physiological/Clinical | Respiratory, thoracic and mediastinal outcomes |
| Trials | Tube tracheotomy | Pulmonary complications | Physiological/Clinical | Respiratory, thoracic and mediastinal outcomes |
| Trials | ARDS | Pulmonary complications | Physiological/Clinical | Respiratory, thoracic and mediastinal outcomes |
| Trials | FEV1(L) | Respiratory function | Physiological/Clinical | Respiratory, thoracic and mediastinal outcomes |
| Trials | FEVC(L) | Respiratory function | Physiological/Clinical | Respiratory, thoracic and mediastinal outcomes |
| Trials | Pulmonary function | Respiratory function | Physiological/Clinical | Respiratory, thoracic and mediastinal outcomes |
| Trials | SaO2 | Respiratory function | Physiological/Clinical | Respiratory, thoracic and mediastinal outcomes |
| Trials | Wound complications | Other Wound Complication | Physiological/Clinical | Skin and subcutaneous tissue outcomes |
| Trials | Wound haematoma | Other Wound Complication | Physiological/Clinical | Skin and subcutaneous tissue outcomes |
| Trials | Wound problem | Other Wound Complication | Physiological/Clinical | Skin and subcutaneous tissue outcomes |
| Trials | Wound seroma | Other Wound Complication | Physiological/Clinical | Skin and subcutaneous tissue outcomes |
| Trials | Incision fat liquefaction | Wound Infection | Physiological/Clinical | Skin and subcutaneous tissue outcomes |
| Trials | Incision infection | Wound Infection | Physiological/Clinical | Skin and subcutaneous tissue outcomes |
| Trials | Wound abscess | Wound Infection | Physiological/Clinical | Skin and subcutaneous tissue outcomes |
| Trials | Wound dehiscence | Wound Infection | Physiological/Clinical | Skin and subcutaneous tissue outcomes |
| Trials | Wound evisceration | Wound Infection | Physiological/Clinical | Skin and subcutaneous tissue outcomes |
| Trials | Wound infection | Wound Infection | Physiological/Clinical | Skin and subcutaneous tissue outcomes |
| Trials | Wound infection/dehiscence | Wound Infection | Physiological/Clinical | Skin and subcutaneous tissue outcomes |
| Trials | Cerebrovascular | Cerebrovascular complications | Physiological/Clinical | Vascular Outcomes |
| Trials | Transient ischemic attack | Cerebrovascular complications | Physiological/Clinical | Vascular Outcomes |
| Trials | Arteriosclerosis obliterans of the leg | Thromboembolic complications | Physiological/Clinical | Vascular Outcomes |
| Trials | Deep vein thrombosis | Thromboembolic complications | Physiological/Clinical | Vascular Outcomes |
| Trials | Pulmonary embolism | Thromboembolic complications | Physiological/Clinical | Vascular Outcomes |
| Trials | Thromboembolic complications | Thromboembolic complications | Physiological/Clinical | Vascular Outcomes |
| Trials | Uncomplicated calf vein thrombosis | Thromboembolic complications | Physiological/Clinical | Vascular Outcomes |
