# Supplementary material for: Core outcome set for surgical trials in gastric cancer (GASTROS study): international patient and healthcare professional consensus
Source: Br J Surg. 2021 Jun 24;108(10):1216–24. doi: 10.1093/bjs/znab192 (PMC10364901; doi:10.1093/bjs/znab192)
Supplement: znab192_Supplementary_Data [file znab192_supplementary_data.zip › Supplementary_file_3_-_Outcome_presented_to_R1_Delphi.docx]

### **Supplementary file 3. Final 56 outcomes presented to participants in round 1 of the Delphi survey.**

| **Outcome** | **Plain language description** | **Domain Area** |
| --- | --- | --- |
| 1. Disease-free survival | How long someone is alive without cancer returning. | Outcomes Related to Death |
| 1. Dying from stomach cancer | Dying from stomach cancer. This does not include dying from treatment for stomach cancer. | Outcomes Related to Death |
| 1. Dying from any cause | Dying from any cause. This includes dying from treatment for stomach cancer. | Outcomes Related to Death |
| 1. Surgery-related death | Dying as a direct consequence of surgery | Outcomes Related to Death |
| 1. Cardiac complications | Complications related to the heart, such as a heart attack or abnormal heart rhythms. | Physiological & Clinical Outcomes |
| 1. Endocrine complications | Complications related to the body’s hormones, such as developing diabetes. | Physiological & Clinical Outcomes |
| 1. Anastomotic complications | Complications related to surgical joins made as a result of removing stomach cancer. | Physiological & Clinical Outcomes |
| 1. Gastro-intestinal functional problems | Symptoms related to how the digestive system works, including those which may become problematic months after discharge from hospital. | Physiological & Clinical Outcomes |
| 1. Bowel Complications | Problems with the bowel, such as those which occur while still in hospital (not including anastomotic complications). | Physiological & Clinical Outcomes |
| 1. Time to recommencing oral intake | The time taken for a patient’s bowel function to return after surgery, such that they can start eating and drinking again. | Physiological & Clinical Outcomes |
| 1. Fatigue | Feeling of tiredness. | Physiological & Clinical Outcomes |
| 1. Multiple organ failure | A severe complication which leads to several organs (such as the heart or lungs) not functioning properly. | Physiological & Clinical Outcomes |
| 1. Pain |  | Physiological & Clinical Outcomes |
| 1. Surgical Stress Response | The body’s response to the stress of surgery. | Physiological & Clinical Outcomes |
| 1. Gallbladder complications | Complications related to the gallbladder. | Physiological & Clinical Outcomes |
| 1. Hepatic Complications | Complications related to the liver. | Physiological & Clinical Outcomes |
| 1. Pancreatic Complications | Complications related to the pancreas. | Physiological & Clinical Outcomes |
| 1. Abdominal Collection | Fluid or infections in the abdomen. | Physiological & Clinical Outcomes |
| 1. Other infections | General infections which are not related to the abdomen, lungs or wounds. | Physiological & Clinical Outcomes |
| 1. Nutritional Effects | The extent to which the body can consume and use the nutrients needed to function properly. | Physiological & Clinical Outcomes |
| 1. Recurrence of Cancer | The chances of the cancer coming back. | Physiological & Clinical Outcomes |
| 1. Renal complications | Complications related to the kidneys, such as kidney failure. | Physiological & Clinical Outcomes |
| 1. Urinary complications | Complications related to the bladder and urinary tract, such as a urinary infection. | Physiological & Clinical Outcomes |
| 1. Post-operative psychosis | A temporary altered mental state after surgery which includes not being able to tell what is or isn’t real. | Physiological & Clinical Outcomes |
| 1. Respiratory complications | Complications such as a chest infection, a collapsed lung or fluid on the lungs. | Physiological & Clinical Outcomes |
| 1. Wound complications | Problems with the surgical incisions, including infection and problems with healing. | Physiological & Clinical Outcomes |
| 1. Cerebro-vascular complications | Complications such as strokes and mini-strokes. | Physiological & Clinical Outcomes |
| 1. Thrombo-embolic complications | Complications such as blood-clots in the legs and lungs. | Physiological & Clinical Outcomes |
| 1. Bleeding | Blood loss as a result of surgery | Physiological & Clinical Outcomes |
| 1. Ability to undertake physical activities | Ability to undertake day-to-day activities including exercise | Life Impact |
| 1. Insomnia | Problems with sleeping. | Life Impact |
| 1. Impact on sexual function | The effect of surgery on a patient’s sexual activity. | Life Impact |
| 1. Ability to eat socially | Ability to eat with friends and family. | Life Impact |
| 1. Ability to interact socially | The ability to have relationships with family and friends. | Life Impact |
| 1. Impact of surgery on social and work roles | The effect of surgery on being able to work and caring for others. | Life Impact |
| 1. Impact on mental health | The effect of surgery on a patient’s psychological well-being. | Life Impact |
| 1. Impact on Physical Appearance | The effect of surgery on a patient’s physical appearance | Life Impact |
| 1. Impact on cognitive functioning | The effect of surgery on concentration and memory. | Life Impact |
| 1. Impact on spirituality or faith | The effect of surgery on a patient’s spirituality or faith. | Life Impact |
| 1. Overall quality of life | An overall measure of how a person’s general wellbeing has been affected by surgery. | Life Impact |
| 1. Impact on perception of physical health | How healthy a patient believes they are following surgery. | Life Impact |
| 1. Ability to complete treatment pathway. | Being well enough to complete all aspects of treatment, such as chemotherapy and/or radiotherapy following surgery. | Life Impact |
| 1. Completeness of tumour removal | Ensuring that the tumour has been surgically removed. | Life Impact |
| 1. Conversion to open surgery | The surgical team having to unexpectedly change the approach from a minimally invasive (laparoscopic or key-hole) operation to a traditional open approach, usually involving a larger incision. | Life Impact |
| 1. Duration of surgery | The length of time taken to perform the surgery. | Life Impact |
| 1. Wound size | The size of the wound or wounds needed to perform the surgery. | Life Impact |
| 1. Cost | The overall cost of surgery. | Resource Use |
| 1. Duration of hospital stay | How long a patient stays in hospital. | Resource Use |
| 1. Readmission to hospital | Whether a patient needs to return to hospital after being discharged following surgery. | Resource Use |
| 1. Destination on Discharge | The location where a patient is discharged to from hospital. | Resource Use |
| 1. Need for an additional intervention. | Unexpected additional procedures or surgeries which may be required. | Resource Use |
| 1. Need for pain relief | The need for a patient to take or be given pain relief after surgery. | Resource Use |
| 1. Duration of stay in an intensive care ward* | How long a patient requires in a critical care or high dependency | Resource Use |
| 1. Adverse drug reaction | Complications related to medications. | Adverse Events |
| 1. All-cause complications | Any complication which may arise after surgery. | Adverse Events |
| 1. Intra-operative complications | Complications which occur during surgery such as accidental injury to an organ. | Adverse Events |
| 1. Anaesthetic complications | Complications specifically related to anaesthesia. | Adverse Events |

*This additional outcome was identified in round 1 and presented to participants in round 2
