# Supplementary material for: Core outcome set for surgical trials in gastric cancer (GASTROS study): international patient and healthcare professional consensus
Source: Br J Surg. 2021 Jun 24;108(10):1216–24. doi: 10.1093/bjs/znab192 (PMC10364901; doi:10.1093/bjs/znab192)
Supplement: znab192_Supplementary_Data [file znab192_supplementary_data.zip › Supplementary_file_4_-_Results_of_Delphi_surveys.docx]

### **Supplementary file 4a. Results of voting after round 1 of the Delphi survey.**

|  | **Patients** | | | **Surgeons** | | | **Nurses** | | |
| --- | --- | --- | --- | --- | --- | --- | --- | --- | --- |
| **Outcome** | **% not important** | **% important** | **% critically important** | **% not important** | **% important** | **% critically important** | **% not important** | **% important** | **% critically important** |
| 1. Disease-free survival | 1.9 | 13.5 | 84.6 | 0.5 | 5.4 | 94.1 | 4.3 | 18.7 | 77.0 |
| 1. Dying from stomach cancer | 2.8 | 11.8 | 85.4 | 0.0 | 6.8 | 93.2 | 2.2 | 15.9 | 81.9 |
| 1. Dying from any cause | 8.1 | 27.8 | 64.1 | 5.0 | 30.1 | 64.8 | 5.6 | 34.2 | 60.2 |
| 1. Surgery-related death | 4.8 | 13.2 | 82.0 | 1.1 | 5.9 | 93.0 | 3.5 | 20.3 | 76.2 |
| 1. Cardiac complications | 8.3 | 29.4 | 62.3 | 5.5 | 45.5 | 49.0 | 4.7 | 34.9 | 60.4 |
| 1. Endocrine complications | 10.9 | 37.7 | 51.5 | 14.0 | 61.9 | 24.1 | 6.0 | 45.7 | 48.3 |
| 1. Anastomotic complications | 5.8 | 19.8 | 74.5 | 0.5 | 4.8 | 94.7 | 1.3 | 16.6 | 82.1 |
| 1. Gastro-intestinal functional problems | 3.0 | 22.4 | 74.5 | 2.1 | 24.7 | 73.3 | 0.0 | 26.8 | 73.2 |
| 1. Bowel Complications | 3.8 | 22.8 | 73.4 | 2.1 | 32.3 | 65.6 | 1.7 | 30.6 | 67.7 |
| 1. Time to recommencing oral intake | 7.3 | 35.8 | 56.9 | 5.5 | 37.3 | 57.3 | 4.2 | 30.9 | 64.8 |
| 1. Fatigue | 13.4 | 35.6 | 51.0 | 11.6 | 58.2 | 30.1 | 12.3 | 49.8 | 37.9 |
| 1. Multiple organ failure | 6.9 | 13.0 | 80.1 | 3.4 | 21.9 | 74.7 | 3.9 | 21.0 | 75.1 |
| 1. Pain | 13.0 | 30.7 | 56.3 | 3.9 | 41.8 | 54.3 | 3.0 | 27.7 | 69.4 |
| 1. Surgical Stress Response | 12.1 | 39.3 | 48.6 | 7.8 | 47.7 | 44.5 | 4.3 | 39.1 | 56.7 |
| 1. Gallbladder complications | 9.3 | 35.4 | 55.3 | 14.4 | 47.9 | 37.7 | 6.9 | 47.2 | 45.9 |
| 1. Hepatic Complications | 8.2 | 28.8 | 63.0 | 10.3 | 49.1 | 40.6 | 4.3 | 43.3 | 52.4 |
| 1. Pancreatic Complications | 8.2 | 24.2 | 67.6 | 5.7 | 35.7 | 58.6 | 3.5 | 40.3 | 56.3 |
| 1. Abdominal Collection | 8.0 | 23.5 | 68.5 | 2.5 | 28.3 | 69.2 | 1.3 | 29.2 | 69.5 |
| 1. Other infections | 9.4 | 30.7 | 59.8 | 4.8 | 41.8 | 53.4 | 3.4 | 31.4 | 65.3 |
| 1. Nutritional Effects | 4.2 | 26.5 | 69.3 | 1.8 | 27.8 | 70.4 | 2.6 | 24.7 | 72.8 |
| 1. Recurrence of Cancer | 5.0 | 4.6 | 90.4 | 0.0 | 4.1 | 95.9 | 0.9 | 15.4 | 83.8 |
| 1. Renal complications | 7.3 | 25.6 | 67.1 | 7.5 | 54.6 | 37.9 | 3.9 | 42.2 | 53.9 |
| 1. Urinary complications | 8.8 | 31.3 | 59.8 | 14.1 | 60.6 | 25.3 | 6.0 | 51.7 | 42.2 |
| 1. Post-operative psychosis | 15.0 | 39.7 | 45.3 | 16.6 | 55.6 | 27.8 | 6.8 | 47.7 | 45.5 |
| 1. Respiratory complications | 8.3 | 23.8 | 67.9 | 2.3 | 38.3 | 59.5 | 2.1 | 28.9 | 68.9 |
| 1. Wound complications | 16.0 | 30.1 | 53.9 | 3.0 | 36.0 | 61.0 | 3.8 | 31.1 | 65.1 |
| 1. Cerebro-vascular complications | 8.6 | 20.2 | 71.2 | 7.3 | 49.2 | 43.5 | 5.1 | 37.2 | 57.7 |
| 1. Thrombo-embolic complications | 8.9 | 16.9 | 74.2 | 3.2 | 36.3 | 60.5 | 3.0 | 30.2 | 66.8 |
| 1. Bleeding | 8.9 | 24.2 | 66.9 | 1.6 | 15.8 | 82.6 | 0.4 | 18.7 | 80.9 |
| 1. Ability to undertake physical activities | 3.8 | 37.6 | 58.6 | 2.3 | 34.0 | 63.7 | 2.2 | 33.9 | 63.9 |
| 1. Insomnia | 19.7 | 44.0 | 36.3 | 15.5 | 60.9 | 23.6 | 7.0 | 47.6 | 45.4 |
| 1. Impact on sexual function | 21.8 | 41.5 | 36.7 | 16.7 | 57.4 | 25.8 | 14.9 | 56.1 | 28.9 |
| 1. Ability to eat socially | 11.8 | 40.3 | 47.9 | 10.2 | 41.6 | 48.1 | 11.0 | 43.6 | 45.4 |
| 1. Ability to interact socially | 13.3 | 37.3 | 49.4 | 10.2 | 45.2 | 44.5 | 8.8 | 44.5 | 46.7 |
| 1. Impact of surgery on social and work roles | 7.3 | 33.7 | 59.0 | 6.8 | 40.7 | 52.6 | 5.3 | 38.6 | 56.1 |
| 1. Impact on mental health | 8.5 | 26.9 | 64.6 | 7.4 | 47.9 | 44.7 | 4.0 | 33.9 | 62.1 |
| 1. Impact on Physical Appearance | 24.7 | 42.2 | 33.1 | 15.8 | 56.7 | 27.4 | 12.3 | 45.8 | 41.9 |
| 1. Impact on cognitive functioning | 10.0 | 29.3 | 60.6 | 11.1 | 49.4 | 39.4 | 8.3 | 44.3 | 47.4 |
| 1. Impact on spirituality or faith | 36.9 | 33.3 | 29.8 | 29.8 | 52.1 | 18.1 | 20.5 | 53.3 | 26.2 |
| 1. Overall quality of life | 4.9 | 23.2 | 71.9 | 1.2 | 18.1 | 80.7 | 2.6 | 26.2 | 71.2 |
| 1. Impact on perception of physical health | 4.2 | 41.6 | 54.2 | 7.7 | 47.4 | 44.9 | 4.4 | 38.2 | 57.5 |
| 1. Ability to complete treatment pathway. | 3.8 | 16.9 | 79.2 | 4.4 | 24.0 | 71.6 | 2.6 | 31.1 | 66.2 |
| 1. Completeness of tumour removal | 3.8 | 4.2 | 92.0 | 0.2 | 5.1 | 94.7 | 0.9 | 14.5 | 84.6 |
| 1. Conversion to open surgery | 23.0 | 24.3 | 52.7 | 11.0 | 29.2 | 59.8 | 6.2 | 32.2 | 61.7 |
| 1. Duration of surgery | 28.2 | 27.5 | 44.3 | 9.5 | 40.9 | 49.5 | 7.5 | 38.3 | 54.2 |
| 1. Wound size | 35.4 | 31.5 | 33.1 | 21.3 | 50.0 | 28.7 | 10.6 | 48.0 | 41.4 |
| 1. Cost | 22.0 | 40.4 | 37.6 | 4.7 | 43.8 | 51.5 | 8.3 | 41.7 | 50.0 |
| 1. Duration of hospital stay | 18.1 | 47.3 | 34.6 | 2.6 | 37.0 | 60.5 | 2.6 | 34.5 | 62.9 |
| 1. Readmission to hospital | 16.3 | 36.4 | 47.3 | 1.4 | 20.3 | 78.3 | 3.5 | 25.8 | 70.7 |
| 1. Destination on Discharge | 25.8 | 41.0 | 33.2 | 13.6 | 45.9 | 40.5 | 16.6 | 47.6 | 35.8 |
| 1. Need for an additional intervention. | 11.8 | 32.5 | 55.7 | 4.0 | 24.8 | 71.2 | 5.2 | 40.9 | 53.9 |
| 1. Need for pain relief | 15.4 | 32.8 | 51.7 | 4.0 | 40.9 | 55.1 | 4.4 | 28.8 | 66.8 |
| 1. Duration of stay in an intensive care ward | Added after round 1 | | | | | | | | |
| 1. Adverse drug reaction | 8.9 | 27.1 | 64.0 | 8.2 | 51.0 | 40.8 | 3.9 | 34.9 | 61.1 |
| 1. All-cause complications | 5.1 | 20.0 | 74.9 | 1.4 | 23.8 | 74.8 | 1.3 | 25.0 | 73.7 |
| 1. Intra-operative complications | 7.6 | 17.1 | 75.3 | 0.5 | 10.2 | 89.3 | 0.9 | 14.4 | 84.7 |
| 1. Anaesthetic complications | 11.0 | 18.5 | 70.5 | 2.6 | 30.3 | 67.1 | 2.2 | 21.1 | 76.7 |

### **Supplementary file 4b. Results of voting after round 2 of the Delphi survey.**

|  | **Patients** | | | **Surgeons** | | | **Nurses** | | |  |
| --- | --- | --- | --- | --- | --- | --- | --- | --- | --- | --- |
| **Outcome** | **% not important** | **% important** | **% critically important** | **% not important** | **% important** | **% critically important** | **% not important** | **% important** | **% critically important** | **Delphi consensus** |
| 1. Disease-free survival | 3.4 | 11.2 | 85.4 | 0.0 | 2.3 | 97.7 | 0.7 | 14.1 | 85.2 | IN |
| 1. Dying from stomach cancer | 2.3 | 11.4 | 86.4 | 0.0 | 3.5 | 96.5 | 1.5 | 18.5 | 80.0 | IN |
| 1. Dying from any cause | 5.8 | 27.5 | 66.7 | 2.6 | 31.9 | 65.5 | 5.2 | 31.3 | 63.4 | NO CONSENSUS |
| 1. Surgery-related death | 2.9 | 13.1 | 84.0 | 0.9 | 2.3 | 96.8 | 3.7 | 18.7 | 77.6 | IN |
| 1. Cardiac complications | 5.7 | 34.7 | 59.7 | 3.5 | 53.1 | 43.4 | 4.4 | 36.3 | 59.3 | NO CONSENSUS |
| 1. Endocrine complications | 8.0 | 47.1 | 44.8 | 9.9 | 74.0 | 16.1 | 7.4 | 51.1 | 41.5 | OUT |
| 1. Anastomotic complications | 2.8 | 20.5 | 76.7 | 0.3 | 4.4 | 95.3 | 0.0 | 15.6 | 84.4 | IN |
| 1. Gastro-intestinal functional problems | 3.3 | 23.9 | 72.8 | 1.2 | 23.9 | 74.9 | 0.0 | 30.4 | 69.6 | NO CONSENSUS |
| 1. Bowel Complications | 3.3 | 24.9 | 71.8 | 1.5 | 37.6 | 60.9 | 2.2 | 37.8 | 60.0 | NO CONSENSUS |
| 1. Time to recommencing oral intake | 7.7 | 43.7 | 48.6 | 4.4 | 39.4 | 56.3 | 6.7 | 33.3 | 60.0 | NO CONSENSUS |
| 1. Fatigue | 13.1 | 46.4 | 40.4 | 9.1 | 62.9 | 28.1 | 8.1 | 60.7 | 31.1 | OUT |
| 1. Multiple organ failure | 3.4 | 10.2 | 86.4 | 1.5 | 17.3 | 81.3 | 1.5 | 18.7 | 79.9 | IN |
| 1. Pain | 12.7 | 33.1 | 54.1 | 1.7 | 42.6 | 55.7 | 3.7 | 37.0 | 59.3 | NO CONSENSUS |
| 1. Surgical Stress Response | 9.7 | 49.1 | 41.1 | 7.0 | 50.3 | 42.7 | 6.0 | 52.6 | 41.4 | OUT |
| 1. Gallbladder complications | 8.2 | 38.6 | 53.2 | 16.7 | 52.9 | 30.4 | 6.8 | 53.8 | 39.4 | NO CONSENSUS |
| 1. Hepatic Complications | 4.6 | 32.9 | 62.4 | 9.3 | 56.6 | 34.1 | 3.0 | 49.6 | 47.4 | NO CONSENSUS |
| 1. Pancreatic Complications | 5.2 | 24.4 | 70.3 | 5.5 | 36.4 | 58.0 | 2.2 | 47.0 | 50.7 | NO CONSENSUS |
| 1. Abdominal Collection | 2.9 | 25.6 | 71.5 | 2.6 | 24.0 | 73.4 | 2.2 | 31.9 | 65.9 | NO CONSENSUS |
| 1. Other infections | 6.3 | 35.6 | 58.0 | 2.6 | 46.9 | 50.4 | 3.0 | 35.8 | 61.2 | NO CONSENSUS |
| 1. Nutritional Effects | 2.7 | 23.5 | 73.8 | 0.9 | 26.3 | 72.8 | 2.2 | 23.0 | 74.8 | IN |
| 1. Recurrence of Cancer | 2.8 | 5.0 | 92.2 | 0.3 | 2.1 | 97.7 | 0.8 | 11.3 | 88.0 | IN |
| 1. Renal complications | 5.3 | 24.7 | 70.0 | 5.3 | 61.4 | 33.3 | 3.8 | 45.9 | 50.4 | NO CONSENSUS |
| 1. Urinary complications | 5.8 | 36.0 | 58.1 | 13.2 | 69.9 | 17.0 | 7.4 | 64.4 | 28.1 | NO CONSENSUS |
| 1. Post-operative psychosis | 12.2 | 48.8 | 39.0 | 14.6 | 63.6 | 21.9 | 5.2 | 60.0 | 34.8 | OUT |
| 1. Respiratory complications | 5.1 | 25.4 | 69.5 | 1.2 | 32.4 | 66.5 | 2.2 | 29.6 | 68.1 | NO CONSENSUS |
| 1. Wound complications | 11.7 | 35.8 | 52.5 | 2.0 | 38.2 | 59.8 | 2.2 | 30.4 | 67.4 | NO CONSENSUS |
| 1. Cerebro-vascular complications | 4.7 | 17.6 | 77.6 | 5.5 | 51.9 | 42.6 | 3.7 | 42.2 | 54.1 | NO CONSENSUS |
| 1. Thrombo-embolic complications | 5.2 | 18.0 | 76.7 | 2.6 | 33.2 | 64.1 | 3.7 | 31.1 | 65.2 | NO CONSENSUS |
| 1. Bleeding | 5.2 | 22.5 | 72.3 | 1.2 | 11.4 | 87.5 | 3.0 | 16.3 | 80.7 | IN |
| 1. Ability to undertake physical activities | 2.2 | 37.4 | 60.4 | 1.5 | 32.1 | 66.5 | 3.0 | 40.7 | 56.3 | NO CONSENSUS |
| 1. Insomnia | 20.3 | 47.3 | 32.4 | 14.0 | 68.8 | 17.2 | 8.1 | 57.0 | 34.8 | OUT |
| 1. Impact on sexual function | 23.0 | 46.0 | 31.0 | 14.6 | 67.0 | 18.4 | 10.4 | 66.7 | 23.0 | OUT |
| 1. Ability to eat socially | 11.5 | 46.2 | 42.3 | 6.1 | 52.8 | 41.1 | 9.6 | 50.4 | 40.0 | OUT |
| 1. Ability to interact socially | 13.2 | 40.7 | 46.2 | 8.2 | 50.1 | 41.7 | 9.6 | 51.9 | 38.5 | OUT |
| 1. Impact of surgery on social and work roles | 8.2 | 40.7 | 51.1 | 4.7 | 43.3 | 52.0 | 5.9 | 45.9 | 48.1 | NO CONSENSUS |
| 1. Impact on mental health | 5.5 | 35.7 | 58.8 | 5.9 | 51.3 | 42.8 | 3.7 | 41.8 | 54.5 | NO CONSENSUS |
| 1. Impact on Physical Appearance | 23.6 | 53.8 | 22.5 | 13.5 | 70.5 | 16.1 | 12.6 | 58.5 | 28.9 | OUT |
| 1. Impact on cognitive functioning | 9.0 | 33.1 | 57.9 | 7.1 | 58.5 | 34.4 | 6.7 | 55.6 | 37.8 | NO CONSENSUS |
| 1. Impact on spirituality or faith | 39.5 | 41.3 | 19.2 | 31.6 | 58.1 | 10.3 | 20.7 | 62.2 | 17.0 | OUT |
| 1. Overall quality of life | 3.9 | 22.1 | 74.0 | 0.6 | 12.9 | 86.5 | 3.0 | 26.7 | 70.4 | IN |
| 1. Impact on perception of physical health | 5.0 | 42.8 | 52.2 | 4.4 | 52.9 | 42.7 | 6.0 | 41.8 | 52.2 | NO CONSENSUS |
| 1. Ability to complete treatment pathway. | 3.9 | 16.3 | 79.8 | 0.6 | 20.8 | 78.6 | 3.0 | 31.1 | 65.9 | NO CONSENSUS |
| 1. Completeness of tumour removal | 2.2 | 5.0 | 92.8 | 0.0 | 2.6 | 97.4 | 0.0 | 12.7 | 87.3 | IN |
| 1. Conversion to open surgery | 20.4 | 28.4 | 51.2 | 10.9 | 30.6 | 58.5 | 6.1 | 30.3 | 63.6 | NO CONSENSUS |
| 1. Duration of surgery | 29.2 | 30.9 | 39.9 | 9.1 | 47.1 | 43.9 | 6.7 | 38.1 | 55.2 | NO CONSENSUS |
| 1. Wound size | 37.0 | 38.7 | 24.3 | 22.5 | 58.2 | 19.3 | 10.4 | 55.2 | 34.3 | OUT |
| 1. Cost | 20.9 | 52.3 | 26.7 | 5.3 | 49.6 | 45.2 | 11.9 | 45.9 | 42.2 | OUT |
| 1. Duration of hospital stay | 20.0 | 51.1 | 28.9 | 2.6 | 40.6 | 56.7 | 3.0 | 36.8 | 60.2 | NO CONSENSUS |
| 1. Readmission to hospital | 15.1 | 35.2 | 49.7 | 0.9 | 20.2 | 78.9 | 0.8 | 29.3 | 69.9 | NO CONSENSUS |
| 1. Destination on Discharge | 25.0 | 46.7 | 28.3 | 9.9 | 55.8 | 34.2 | 18.0 | 51.1 | 30.8 | OUT |
| 1. Need for an additional intervention. | 10.2 | 33.9 | 55.9 | 1.5 | 23.1 | 75.4 | 5.2 | 38.1 | 56.7 | NO CONSENSUS |
| 1. Need for pain relief | 14.5 | 35.2 | 50.3 | 2.9 | 42.7 | 54.4 | 3.8 | 27.8 | 68.4 | NO CONSENSUS |
| 1. Duration of stay in an intensive care ward | 2.9 | 32.9 | 64.1 | 1.2 | 43.2 | 55.7 | 2.3 | 35.2 | 62.5 | NO CONSENSUS |
| 1. Adverse drug reaction | 6.7 | 26.3 | 67.0 | 8.2 | 55.3 | 36.5 | 6.2 | 36.2 | 57.7 | NO CONSENSUS |
| 1. All-cause complications | 3.9 | 20.2 | 75.8 | 1.2 | 17.6 | 81.2 | 1.5 | 20.6 | 77.9 | IN |
| 1. Intra-operative complications | 6.3 | 13.1 | 80.6 | 0.3 | 8.2 | 91.5 | 0.0 | 14.6 | 85.4 | IN |
| 1. Anaesthetic complications | 7.4 | 17.7 | 74.9 | 1.5 | 28.0 | 70.5 | 2.4 | 19.7 | 78.0 | IN |
