# Supplementary material for: Core outcome set for surgical trials in gastric cancer (GASTROS study): international patient and healthcare professional consensus
Source: Br J Surg. 2021 Jun 24;108(10):1216–24. doi: 10.1093/bjs/znab192 (PMC10364901; doi:10.1093/bjs/znab192)
Supplement: znab192_Supplementary_Data [file znab192_supplementary_data.zip › Supplementary_file_5_-_Additional_suggested_outcomes.docx]

### **Supplementary file 5. Suggested ‘additional’ outcomes from round 1 Delphi survey participants to consider for presentation in round 2. *These suggestions were grouped into one outcome -‘duration of stay in an intensive care ward’. **SMG= study management group.**

| **Suggestion by Delphi participant (verbatim and translated)** | **Reason for inclusion or exclusion** | **Which Domain is this already included in?** | **SMG** Decision** | **Reviewer Decision** | **Final Decision** |
| --- | --- | --- | --- | --- | --- |
| *Critical care utilisation | New outcome | N/A | Include | Include | **Include** |
| *Duration of ICU Admission | New outcome | N/A | Include | Include | **Include** |
| *Intensive Care duration | New outcome | N/A | Include | Include | **Include** |
| Adverse effect on patients spouse; how they cope | Ambiguous term | N/A | Exclude | Exclude | Exclude |
| Family support | Ambiguous term | N/A | Exclude | Exclude | Exclude |
| Positive? | Ambiguous term | N/A | Exclude | Exclude | Exclude |
| Reviews | Ambiguous term | N/A | Exclude | Exclude | Exclude |
| Access route for surgery (ex open; laparoscopic; robotic) | Not an outcome | N/A | Exclude | Exclude | Exclude |
| Adherence to trial protocol | Not an outcome | N/A | Exclude | Exclude | Exclude |
| Being fully informed as regard to the disease | Not an outcome | N/A | Exclude | Exclude | Exclude |
| Blocking of plexus via endoscopic ultrasound | Not an outcome | N/A | Exclude | Exclude | Exclude |
| Compliance of the surgery | Not an outcome | N/A | Exclude | Exclude | Exclude |
| cTNM | Not an outcome | N/A | Exclude | Exclude | Exclude |
| Finding of CTM positive to pre-neoadivative exploratory laparoscopy | Not an outcome | N/A | Exclude | Exclude | Exclude |
| Gastric resection (distal gastrectomy, total gastrectomy, proximal gastrectomy) | Not an outcome | N/A | Exclude | Exclude | Exclude |
| General health/performance status before treatment | Not an outcome | N/A | Exclude | Exclude | Exclude |
| Geographical location of study | Not an outcome | N/A | Exclude | Exclude | Exclude |
| Importance of endoscopic follow-up | Not an outcome | N/A | Exclude | Exclude | Exclude |
| Laparoscopic-endoscopic procedures (LECS) | Not an outcome | N/A | Exclude | Exclude | Exclude |
| Learning curve for procedure / surgeon experience | Not an outcome | N/A | Exclude | Exclude | Exclude |
| Number of patients receiving Neo-adjuvant therapy | Not an outcome | N/A | Exclude | Exclude | Exclude |
| Number of patients undergoing bi-directional chemotherapy during surgery | Not an outcome | N/A | Exclude | Exclude | Exclude |
| Number of patients undergoing HIPEC hyperthermic intraperitoneal chemotherapy | Not an outcome | N/A | Exclude | Exclude | Exclude |
| Operator qualification experience | Not an outcome | N/A | Exclude | Exclude | Exclude |
| Organ-preserving operations in gastric cancer surgery | Not an outcome | N/A | Exclude | Exclude | Exclude |
| Percentage of Stomach Removed | Not an outcome | N/A | Exclude | Exclude | Exclude |
| Possibility to participate in special study or experimental treatment (eg immunotherapy) | Not an outcome | N/A | Exclude | Exclude | Exclude |
| Post operative advice & support | Not an outcome | N/A | Exclude | Exclude | Exclude |
| Post treatment monitoring (scans; blood tests; endoscopy; etc). | Not an outcome | N/A | Exclude | Exclude | Exclude |
| Pre-surgical considerations such as dental treatment not possible when on Apixaban | Not an outcome | N/A | Exclude | Exclude | Exclude |
| Reconstruction method (Bi I, Bi II, ROUX-Y) | Not an outcome | N/A | Exclude | Exclude | Exclude |
| Simultaneous treatment of liver metastases | Not an outcome | N/A | Exclude | Exclude | Exclude |
| Surgical approach | Not an outcome | N/A | Exclude | Exclude | Exclude |
| T is important that a hospital takes care of the patient from the beginning to the end and that all the tests are done in the hospital and do not leave the patient at the mercy of the cup !!!!!! | Not an outcome | N/A | Exclude | Exclude | Exclude |
| The most important thing is the patient's trust to the doctor and the team | Not an outcome | N/A | Exclude | Exclude | Exclude |
| Time from first symptom to time of medical consultation | Not an outcome | N/A | Exclude | Exclude | Exclude |
| Travel time to treatment center | Not an outcome | N/A | Exclude | Exclude | Exclude |
| Tumour histology | Not an outcome | N/A | Exclude | Exclude | Exclude |
| Tumour site | Not an outcome | N/A | Exclude | Exclude | Exclude |
| Use of drainage | Not an outcome | N/A | Exclude | Exclude | Exclude |
| Complications of adjuvant treatment | Not an outcome related to surgery | N/A | Exclude | Exclude | Exclude |
| Complications of neoadjuvant treatment | Not an outcome related to surgery | N/A | Exclude | Exclude | Exclude |
| Comprehensive treatment | Not an outcome related to surgery | N/A | Exclude | Exclude | Exclude |
| Hospitalization satisfaction, patient hospitalization experience | Not an outcome related to surgery | N/A | Exclude | Exclude | Exclude |
| Neoadjuvant versus adjuvant chemotherapy (perioperative complications; DFS; OS) | Not an outcome related to surgery | N/A | Exclude | Exclude | Exclude |
| Neoadjuvant/adjuvant modality treatment | Not an outcome related to surgery | N/A | Exclude | Exclude | Exclude |
| Patient satisfaction | Not an outcome related to surgery | N/A | Exclude | Exclude | Exclude |
| (Duodeno)gastro-esophageal reflux | Outcome already presented in round 1 | Gastro-intestinal functional problems | Exclude | Exclude | Exclude |
| Ability to eat (rather than just socially) | Outcome already presented in round 1 | Gastro-intestinal functional problems | Exclude | Exclude | Exclude |
| Ability to perform physical activity/sports | Outcome already presented in round 1 | Ability to undertake physical activities | Exclude | Exclude | Exclude |
| Ability to resume eating to get enough calories to maintain a healthy life | Outcome already presented in round 1 | Nutritional Effects | Exclude | Exclude | Exclude |
| Amount of ingested meal compared to before gastrectomy | Outcome already presented in round 1 | Multiple | Exclude | Exclude | Exclude |
| Anemia | Outcome already presented in round 1 | Bleeding | Exclude | Exclude | Exclude |
| Anorexia | Outcome already presented in round 1 | Gastro-intestinal functional problems | Exclude | Exclude | Exclude |
| BMI | Outcome already presented in round 1 | Nutritional Effects | Exclude | Exclude | Exclude |
| Body shape(weight change) | Outcome already presented in round 1 | Nutritional Effects | Exclude | Exclude | Exclude |
| Change in body weight | Outcome already presented in round 1 | Nutritional Effects | Exclude | Exclude | Exclude |
| Changes of body weight 1;3;6;12 months after gastrectomy | Outcome already presented in round 1 | Nutritional Effects | Exclude | Exclude | Exclude |
| Degree of radical cure (palliative, radical) | Outcome already presented in round 1 | Completeness of tumour removal | Exclude | Exclude | Exclude |
| Details of neo-adjuvant chemotherapy | Outcome already presented in round 1 | Ability to complete treatment pathway. | Exclude | Exclude | Exclude |
| Development of dumping syndrome | Outcome already presented in round 1 | Gastro-intestinal functional problems | Exclude | Exclude | Exclude |
| Difference of postgastrectomy diet (english; continental; korean; chinese) | Outcome already presented in round 1 | Multiple | Exclude | Exclude | Exclude |
| Discomfort; calmness ; panic | Outcome already presented in round 1 | Pain | Exclude | Exclude | Exclude |
| Drainage withdrawal day | Outcome already presented in round 1 | Multiple | Exclude | Exclude | Exclude |
| Dumping syndrome | Outcome already presented in round 1 | Gastro-intestinal functional problems | Exclude | Exclude | Exclude |
| Dumping syndrome - impact on day-to-day functioning | Outcome already presented in round 1 | Gastro-intestinal functional problems | Exclude | Exclude | Exclude |
| Dumping years after surgery | Outcome already presented in round 1 | Gastro-intestinal functional problems | Exclude | Exclude | Exclude |
| Early and late dumping | Outcome already presented in round 1 | Gastro-intestinal functional problems | Exclude | Exclude | Exclude |
| Early dumping abdominal symptoms | Outcome already presented in round 1 | Gastro-intestinal functional problems | Exclude | Exclude | Exclude |
| Early dumping general symptoms | Outcome already presented in round 1 | Gastro-intestinal functional problems | Exclude | Exclude | Exclude |
| Eating | Outcome already presented in round 1 | Nutritional Effects | Exclude | Exclude | Exclude |
| Effect of treatment on memory | Outcome already presented in round 1 | Impact on cognitive functioning | Exclude | Exclude | Exclude |
| Effect of treatment on relationship | Outcome already presented in round 1 | Ability to interact socially | Exclude | Exclude | Exclude |
| Effects of alcohol consumption | Outcome already presented in round 1 | Nutritional Effects | Exclude | Exclude | Exclude |
| Effects of fizzy drinks | Outcome already presented in round 1 | Gastro-intestinal functional problems | Exclude | Exclude | Exclude |
| Effects on metabolic diseases such as hypertension, diabetes, and gout | Outcome already presented in round 1 | Multiple | Exclude | Exclude | Exclude |
| Exocrine complications e.g. Pancreatic insufficiency | Outcome already presented in round 1 | Pancreas complications | Exclude | Exclude | Exclude |
| Fatigue | Outcome already presented in round 1 | Fatigue | Exclude | Exclude | Exclude |
| Functional outcome related to different type of reconstruction after gastrectomy | Outcome already presented in round 1 | Gastro-intestinal functional problems | Exclude | Exclude | Exclude |
| Gain a useful life to provide for dependants & self or lose life in the attempt | Outcome already presented in round 1 | Impact of surgery on social and work roles | Exclude | Exclude | Exclude |
| Hematologic complications (mainly anemia) | Outcome already presented in round 1 | Bleeding | Exclude | Exclude | Exclude |
| Household income | Outcome already presented in round 1 | Impact of surgery on social and work roles | Exclude | Exclude | Exclude |
| How your body responded after the surgery. | Outcome already presented in round 1 | Surgical Stress Response | Exclude | Exclude | Exclude |
| Impact of dumping syndrome | Outcome already presented in round 1 | Gastro-intestinal functional problems | Exclude | Exclude | Exclude |
| Impact of treatment on weight | Outcome already presented in round 1 | Nutritional Effects | Exclude | Exclude | Exclude |
| In hospital mortality | Outcome already presented in round 1 | Surgery-related death | Exclude | Exclude | Exclude |
| Incidence of bile acid malabsorption | Outcome already presented in round 1 | Gastro-intestinal functional problems | Exclude | Exclude | Exclude |
| Incidence of small intestinal bacterial overgrowth | Outcome already presented in round 1 | Gastro-intestinal functional problems | Exclude | Exclude | Exclude |
| Ingested amount of food per day | Outcome already presented in round 1 | Nutritional Effects | Exclude | Exclude | Exclude |
| Late dumping symptoms | Outcome already presented in round 1 | Gastro-intestinal functional problems | Exclude | Exclude | Exclude |
| Length of absenteeism from work | Outcome already presented in round 1 | Impact of surgery on social and work roles | Exclude | Exclude | Exclude |
| Length of Recovery time | Outcome already presented in round 1 | Duration of Hospital Stay | Exclude | Exclude | Exclude |
| Life or death decisions/risks are vitally important. Quality of life effects if only temporary are important to be aware of; but not so important. Those side effects of surgery which are going to be permanent are also critically important for the patient to know about and the level of risk. They can then make an informed decision and be more mentally prepared for the process as it unfolds and also be able to manage their conditions in the longer term. | Outcome already presented in round 1 | Overall Quality of Life | Exclude | Exclude | Exclude |
| Lost of the job | Outcome already presented in round 1 | Impact of surgery on social and work roles | Exclude | Exclude | Exclude |
| Lost of the partner | Outcome already presented in round 1 | Ability to interact socially  - The ability to have relationships with family and friends. | Exclude | Exclude | Exclude |
| Lymph node cleaning range (D1, D2, D2+, D3) | Outcome already presented in round 1 | Completeness of tumour removal | Exclude | Exclude | Exclude |
| More about nutritional outcome | Outcome already presented in round 1 | Nutritional Effects | Exclude | Exclude | Exclude |
| Multivisceral resection | Outcome already presented in round 1 | Completeness of tumour removal | Exclude | Exclude | Exclude |
| Muscle function (physical activity) | Outcome already presented in round 1 | Nutritional Effects | Exclude | Exclude | Exclude |
| Nausea | Outcome already presented in round 1 | Gastro-intestinal functional problems | Exclude | Exclude | Exclude |
| Nausea and vomiting | Outcome already presented in round 1 | Gastro-intestinal functional problems | Exclude | Exclude | Exclude |
| Need for a dietitian after discharge | Outcome already presented in round 1 | Nutritional Effects | Exclude | Exclude | Exclude |
| Need for supplementary feeding | Outcome already presented in round 1 | Nutritional Effects | Exclude | Exclude | Exclude |
| No appetite | Outcome already presented in round 1 | Gastro-intestinal functional problems | Exclude | Exclude | Exclude |
| Number of patients receiving Adjuvant therapy | Outcome already presented in round 1 | Ability to complete treatment pathway. | Exclude | Exclude | Exclude |
| Number of resected lymph nodes | Outcome already presented in round 1 | Completeness of tumour removal | Exclude | Exclude | Exclude |
| Nutritional condition improving measures (e.g., PE) | Outcome already presented in round 1 | Nutritional Effects | Exclude | Exclude | Exclude |
| Nutritional support by endoscopy (probes, prostheses, dilatations) | Outcome already presented in round 1 | Need for an additional intervention. | Exclude | Exclude | Exclude |
| Operation time | Outcome already presented in round 1 | Duration of surgery | Exclude | Exclude | Exclude |
| Other treatments after surgery | Outcome already presented in round 1 | Ability to complete treatment pathway. | Exclude | Exclude | Exclude |
| Overall survival e disease free survival post HIPEC | Outcome already presented in round 1 | Overall survival | Exclude | Exclude | Exclude |
| Palliative resection | Outcome already presented in round 1 | Completeness of tumour removal | Exclude | Exclude | Exclude |
| Patients with peritoneal recurrence of post-HIPEC gastric cancer | Outcome already presented in round 1 | Recurrence of Cancer | Exclude | Exclude | Exclude |
| Percentage weight-loss. | Outcome already presented in round 1 | Nutritional Effects | Exclude | Exclude | Exclude |
| Permanent pain after treatment | Outcome already presented in round 1 | Pain | Exclude | Exclude | Exclude |
| Post op histology | Outcome already presented in round 1 | Completeness of tumour removal | Exclude | Exclude | Exclude |
| Post operative inability to eat normally. Weight loss and further care needed. | Outcome already presented in round 1 | Nutritional Effects | Exclude | Exclude | Exclude |
| Postoperative considerations and post-treatment planning and programs. | Outcome already presented in round 1 | Ability to complete treatment pathway. | Exclude | Exclude | Exclude |
| Postprandial fullness | Outcome already presented in round 1 | Gastro-intestinal functional problems | Exclude | Exclude | Exclude |
| Post-surgical nutrition | Outcome already presented in round 1 | Nutritional Effects | Exclude | Exclude | Exclude |
| Prevalence of dumping syndrome | Outcome already presented in round 1 | Gastro-intestinal functional problems | Exclude | Exclude | Exclude |
| pTNM | Outcome already presented in round 1 | Completeness of tumour removal | Exclude | Exclude | Exclude |
| Quality of resection specimen and pathology outcomes | Outcome already presented in round 1 | Completeness of tumour removal | Exclude | Exclude | Exclude |
| Quality of surgery | Outcome already presented in round 1 | Completeness of tumour removal | Exclude | Exclude | Exclude |
| Realisation of how life would change after the operation | Outcome already presented in round 1 | Overall Quality of Life | Exclude | Exclude | Exclude |
| Recurrence place (liver vs peritoneum vs lymph node vs lungs vs others) | Outcome already presented in round 1 | Recurrence of Cancer | Exclude | Exclude | Exclude |
| Regurgitation | Outcome already presented in round 1 | Gastro-intestinal functional problems | Exclude | Exclude | Exclude |
| Reoperation cause | Outcome already presented in round 1 | Need for an additional intervention. | Exclude | Exclude | Exclude |
| Restoration of bowel function | Outcome already presented in round 1 | Time to recommencing oral intake | Exclude | Exclude | Exclude |
| Retinal sac resection (complete, incomplete, unremoved) | Outcome already presented in round 1 | Completeness of tumour removal | Exclude | Exclude | Exclude |
| Sarcopenia | Outcome already presented in round 1 | Nutritional Effects | Exclude | Exclude | Exclude |
| Severity of various symptoms after gastrectomy | Outcome already presented in round 1 | Multiple | Exclude | Exclude | Exclude |
| Site of recurrence | Outcome already presented in round 1 | Recurrence of Cancer | Exclude | Exclude | Exclude |
| Start of liquid intake | Outcome already presented in round 1 | Time to recommencing oral intake | Exclude | Exclude | Exclude |
| Start of solid diet | Outcome already presented in round 1 | Time to recommencing oral intake | Exclude | Exclude | Exclude |
| Sweeping lymph node group | Outcome already presented in round 1 | Completeness of tumour removal | Exclude | Exclude | Exclude |
| Sweeping the number of lymph nodes | Outcome already presented in round 1 | Completeness of tumour removal | Exclude | Exclude | Exclude |
| Time of recurrence | Outcome already presented in round 1 | Recurrence of Cancer | Exclude | Exclude | Exclude |
| Time spent in the hospital (surgery+chemo+artificial nutrition+palliative etc. Etc.) | Outcome already presented in round 1 | Duration of Hospital Stay | Exclude | Exclude | Exclude |
| Time to and percentage of patients with chemotherapy initiation | Outcome already presented in round 1 | Ability to complete treatment pathway. | Exclude | Exclude | Exclude |
| Time to mobilization | Outcome already presented in round 1 | Ability to undertake physical activities | Exclude | Exclude | Exclude |
| Tiredness just after mild efforts it’s really considerable; I have a limited autonomy during the day | Outcome already presented in round 1 | Fatigue | Exclude | Exclude | Exclude |
| Weight loss | Outcome already presented in round 1 | Nutritional Effects | Exclude | Exclude | Exclude |
| Weight loss after surgery | Outcome already presented in round 1 | Nutritional Effects | Exclude | Exclude | Exclude |
| 12 month survival | Relates to how or when an outcome is measured | N/A | Exclude | Exclude | Exclude |
| 90 day survival | Relates to how or when an outcome is measured | N/A | Exclude | Exclude | Exclude |
| PROMS at 3,6&12 months | Relates to how or when an outcome is measured | N/A | Exclude | Exclude | Exclude |
