# Supplementary material for: Core outcome set for surgical trials in gastric cancer (GASTROS study): international patient and healthcare professional consensus
Source: Br J Surg. 2021 Jun 24;108(10):1216–24. doi: 10.1093/bjs/znab192 (PMC10364901; doi:10.1093/bjs/znab192)
Supplement: znab192_Supplementary_Data [file znab192_supplementary_data.zip › Supplementary_file_6_-_Consensus_meeting_results.docx]

### **Supplementary file 6. Summary and results from the GASTROS Consensus meeting held on Sunday the 8^th^ of March 2020.**

**Participant information**

- 43 participants (7 patients ,7 nurses, 29 surgeons)
- 14 countries (4 continents – South America, North America, Europe, Asia).
  - 1 patient from Netherlands (remainder from UK)
  - 1 nurse from Belgium (remainder from UK)
  - 20 surgeons from Europe (9 UK), 3 from South America, 2 North America, 1 East Asia, 3 Central Asia.
- 18 in Manchester; 25 online
- 40 participants were present throughout the entire day. Three left halfway through the meeting (2 from the venue and 1 online).
  - 21 online had intermittent technical problems with voting app but made a note of their votes and supplied them on an online form with 24 hours of the meeting.

**Complications**

The first session discussed how complications should be reported. This was required because ‘all-cause complications’ had reached consensus to include in the core outcome set based on the Delphi survey, whilst some complications were excluded. To record ‘all-cause’ complications, all complications must be recorded as a minimum. To clarify this potential disparity, participants were asked to discuss different options and vote on their preference. After two rounds of voting, there was no clear consensus how complications should be reported. This has identified the need for further work on this topic. For the manuscript, the term ‘complications’ will be used and qualified by explaining that more work is required.

The remaining outcomes were discussed and rated by participants. No further outcomes were added to the core outcome set. The following charts illustrate how participants voted during the meeting.

**Definitions**

Participants were asked how definitions should be finalised. The majority of participants agreed that this should be a process which involves both healthcare professionals and patients.

**Results from consensus meeting voting related to non-complication type outcomes.**

**Results from voting related to who should be involved in defining outcomes.**
